# CIBRA identifies genomic alterations with a system-wide impact on tumor biology

**DOI:** 10.1093/bioinformatics/btae384

**Published:** 2024-09-04

**Authors:** Soufyan Lakbir, Caterina Buranelli, Gerrit A Meijer, Jaap Heringa, Remond J A Fijneman, Sanne Abeln

**Affiliations:** Bioinformatics Section, Department of Computer Science, Vrije Universiteit Amsterdam, Amsterdam, The Netherlands; Translational Gastrointestinal Oncology Group, Department of Pathology, Netherlands Cancer Institute, Amsterdam, The Netherlands; AI Technology for Life group, Department of Information and Computing Sciences and Department of Biology, Utrecht University, Utrecht, The Netherlands; Bioinformatics Section, Department of Computer Science, Vrije Universiteit Amsterdam, Amsterdam, The Netherlands; Translational Gastrointestinal Oncology Group, Department of Pathology, Netherlands Cancer Institute, Amsterdam, The Netherlands; Translational Gastrointestinal Oncology Group, Department of Pathology, Netherlands Cancer Institute, Amsterdam, The Netherlands; Bioinformatics Section, Department of Computer Science, Vrije Universiteit Amsterdam, Amsterdam, The Netherlands; Translational Gastrointestinal Oncology Group, Department of Pathology, Netherlands Cancer Institute, Amsterdam, The Netherlands; Bioinformatics Section, Department of Computer Science, Vrije Universiteit Amsterdam, Amsterdam, The Netherlands; AI Technology for Life group, Department of Information and Computing Sciences and Department of Biology, Utrecht University, Utrecht, The Netherlands

## Abstract

**Motivation:**

Genomic instability is a hallmark of cancer, leading to many somatic alterations. Identifying which alterations have a system-wide impact is a challenging task. Nevertheless, this is an essential first step for prioritizing potential biomarkers. We developed CIBRA (Computational Identification of Biologically Relevant Alterations), a method that determines the system-wide impact of genomic alterations on tumor biology by integrating two distinct omics data types: one indicating genomic alterations (e.g. genomics), and another defining a system-wide expression response (e.g. transcriptomics). CIBRA was evaluated with genome-wide screens in 33 cancer types using primary and metastatic cancer data from the Cancer Genome Atlas and Hartwig Medical Foundation.

**Results:**

We demonstrate the capability of CIBRA by successfully confirming the impact of point mutations in experimentally validated oncogenes and tumor suppressor genes (0.79 AUC). Surprisingly, many genes affected by structural variants were identified to have a strong system-wide impact (30.3%), suggesting that their role in cancer development has thus far been largely under-reported. Additionally, CIBRA can identify impact with only 10 cases and controls, providing a novel way to prioritize genomic alterations with a prominent role in cancer biology. Our findings demonstrate that CIBRA can identify cancer drivers by combining genomics and transcriptomics data. Moreover, our work shows an unexpected substantial system-wide impact of structural variants in cancer. Hence, CIBRA has the potential to preselect and refine current definitions of genomic alterations to derive more nuanced biomarkers for diagnostics, disease progression, and treatment response.

**Availability and implementation:**

The R package CIBRA is available at https://github.com/AIT4LIFE-UU/CIBRA.

## 1 Introduction

Cancer is characterized by genomic instability leading to many somatic alterations, ranging from single nucleotide variants (SNVs) to large-scale somatic copy number aberrations (SCNAs) and structural variants (SVs) (Hanahan and Weinberg 2011). While the majority of alterations have no defined impact on tumor biology, a few alterations contribute to the development and progression of cancer (Martínez-Jiménez *et al.* 2020). Computationally identifying these key somatic alterations with a major impact on tumor biology is challenging (Porta-Pardo *et al.* 2017, Martínez-Jiménez *et al.* 2020, Ostroverkhova *et al.* 2023). Generally, there are two types of computational approaches for identifying biologically relevant alterations: frequency-based methods and impact prediction methods.

Frequency-based methods such as MutSigCV (Lawrence *et al.* 2013), OncodriveFM (Gonzalez-Perez and Lopez-Bigas 2012), OncodriveCLUST (Arnedo-Pac *et al.* 2019), and MutSig-CL (Lawrence *et al.* 2014) identify biologically relevant alterations through enrichment of alterations, for example, at the population level or sublocalized within a protein (Niu *et al.* 2016, Porta-Pardo *et al.* 2017). They rely on the rationale that tumorigenesis follows a Darwinian evolution characterized by variation and selection (Porta-Pardo *et al.* 2017, Martínez-Jiménez *et al.* 2020). However, frequency-based methods rely on large cohorts to identify biologically relevant alterations. Moreover, replication timing, chromatin structure, methylation status, or low-complexity regions can all influence the rate of alterations throughout the genome, leading to hot spots, such as fragile sites for SVs. Due to these factors, low-frequency alterations and SVs pose a challenge for frequency-based methods (Sherman *et al.* 2022, Ostroverkhova et al. 2023).

Impact prediction methods such as SIFT (Ng and Henikoff 2003), PolyPhen (Adzhubei *et al.* 2010), or the more recent AlphaMissense (Cheng *et al.* 2023) can effectively estimate the potential impact of variants on the protein and, its pathogenicity. However, these methods can only assess the impact of missense SNVs within a coding region, making it impossible to assess the impact of, for example, SVs and non-coding variants.

In this work, we explore the idea of using gene expression levels to systematically assess the biological impact of genomic alterations; this approach can address many of the shortcomings listed above. The transcriptome is a phenotypic representation of the cellular system, which can reflect the status of cellular processes, as well as tissue composition (Guinney *et al.* 2014, Knijnenburg *et al.* 2018, Way *et al.* 2018). The key idea is to observe if a genomic alteration is associated with a system-wide change in gene expression levels. Previously, we have shown that transcriptomics data can be used to predict system-wide changes, such as genomic instability, by training a random forest model to predict the tumor break load (Lakbir *et al.* 2022). Moreover, the work from Crawford et al. (2022) and Aben *et al.* (2018) highlighted that among omics data modalities, the transcriptome can most closely reflect the system-wide change caused by genomic alterations (Aben *et al.* 2018, Crawford et al. 2022). As such, we hypothesize that biologically relevant genomic alterations elicit characteristic changes through the system, reflecting the genomic change, whereas alterations without an impact will have no defined systemic change on the system. In this study, we introduce a novel computational method, CIBRA (Computational Identification of Biologically Relevant Alterations), that identifies the system-wide impact of genomic alterations by integrating genomics with transcriptomics data. By assessing the degree of change in the system, we can determine the extent of impact of a genomic alteration. This approach can complement current methods in identifying the impact of low-frequency alterations and SVs, which can aid our understanding of cancer biology, consequences for clinical behavior, and ultimately personalized care (van Belzen *et al.* 2021, Doig *et al.* 2022).

## 2 Materials and methods

First, to validate if CIBRA can identify known tumor suppressor genes and oncogenes, we performed a pan-cancer genome-wide screen of coding SNVs in primary cancers. Next, to assess the impact of SVs in cancer, we conducted a genome-wide screen on genes affected by SVs in metastatic colorectal, breast, and lung cancer. In addition, we explored the capability of CIBRA to identify the most impactful type of alteration and gene subregion of known oncogenes and tumor suppressor genes. Lastly, we show an additional utility of CIBRA, the similarity score, that assesses the categorical similarity in terms of the observed system-wide impact between different variants within the same gene, or between different genes.

### 2.1 CIBRA scores

**Figure 1.**
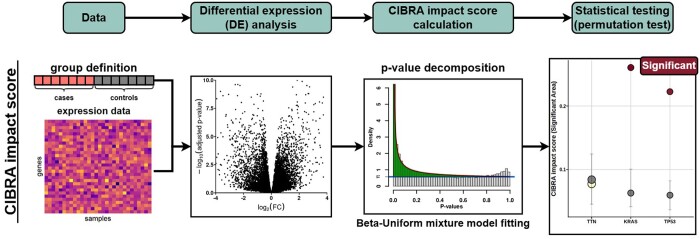
Flowchart of the CIBRA impact score calculation. As input CIBRA takes expression data and a group definition in terms of cases and controls: for example, mutated versus WT samples for a specific genomic alteration. These inputs are used to perform differential expression (DE) analysis between the cases and controls. The *P*-value distribution generated from the DE analysis is subsequently decomposed by fitting a Beta–Uniform mixture model. From the model, the CIBRA impact score termed the significant area is calculated by taking the integral between the Beta and Uniform components of the mixture model, depicted as the green area in the third panel. Finally, the statistical significance of the impact measure is assessed with a permutation test.

To infer the system-wide impact of genomic alterations, CIBRA provides the CIBRA impact score (Fig. 1). The impact score reflects the fraction of genes with altered expression, representing the system-wide impact of a genomic alteration. The similarity score represents the similarity between two conditions in terms of their system-wide effect. CIBRA probes system-wide responses based on samples with (cases) and without (controls) genomic alterations using a Beta–Uniform mixture model (Pounds and Morris 2003) to decompose the *P*-value distribution generated from differential expression (DE) analysis. For the CIBRA impact score, the significant area between the Beta and Uniform components of the Beta–Uniform mixture model is calculated. The significant area is the integral between the Beta and Uniform components calculated as:

(1) $$\int_{0}^{1}f(x)dx=\alpha (1-\lambda )(x^{\alpha -1}-1)\;\mathrm{for}\;\lambda ,\alpha \;\in \;(0,1)$$

where *λ* and *α* are the estimated shape parameters of the Beta–Uniform mixture model. The significant area indicates the extent of *P*-values that arise from the alternative component, indicating the extent of change in the system. Under the null hypothesis of no system-wide change, the significant area is expected to be 0, while it increases with an increasing change in the system up to a theoretical maximum of 1 (Fig. 1). The significant area is the measure that describes the extent of the system-wide impact and will be referred to as the CIBRA impact score in this manuscript. An additional measure is derived to accommodate and detect biases in the *P*-value distribution as described in the Supplementary materials and methods. The statistical significance of the impact measures is assessed by performing 1000 sample permutations and assessing the observed impact score with respect to the permutation distribution (Fig. 1).

To compare the system-wide impact between genomic alterations, we have derived the CIBRA similarity score (Supplementary Fig. S1). For the similarity score, group definitions with shared controls are used to perform differential expression analysis. The generated *log*_2_ fold changes and adjusted *P*-values are used to define DE states. These states assign genes in whether they are significantly up- or down-regulated or if they are not significantly changed. From the list of DE states, a similarity and anti-similarity score is calculated between the conditions (Supplementary Fig. S1). The significance of the scores is assessed with a permutation test. A full description of the methodology is described in the Supplementary materials and methods.

### 2.2 Machine learning

To assess if the impact of a genomic alteration could also be investigated by a machine learning model, we trained a random forest model that predicts the genomic alteration status from transcriptomics data. In this work, transcriptomics data has been used as a measure of the system changes. Full details of the machine learning model are described in the Supplementary materials and methods.

### 2.3 Data

Public data from The Cancer Genome Atlas (TCGA) were gathered from the Genome Data Commons (GDC) portal for 33 cancer types (Grossman *et al.* 2016). Clinical data and tumor mutational burden (TMB) were retrieved from cBioPortal (Weinstein *et al.* 2013). “Silent” variants indicated by the variant classification provided in the Mutation Annotation Format (MAF) file were removed from the genome-wide screen analysis. From the Hartwig Medical Foundation (HMF), whole genome sequencing (WGS) data was retrieved from 610 metastatic colorectal cancer samples, 996 metastatic breast cancer samples, and 551 metastatic lung cancer samples (Priestley *et al.* 2019). RNA sequencing data was available from 394 metastatic colorectal cancer samples, 332 metastatic breast cancer samples, and 127 metastatic lung cancer samples. “Silent” variants indicated by the SnpEff (version 4.3, RRID: SCR_005191) canonical transcript summary were removed from the SNV calls in all further analyses that indicated coding variants. SV calls were only retained when they passed all filters from the HMF pipeline.

## 3 Results

### 3.1 CIBRA identifies the system-wide impact of known cancer genes

**Figure 2.**
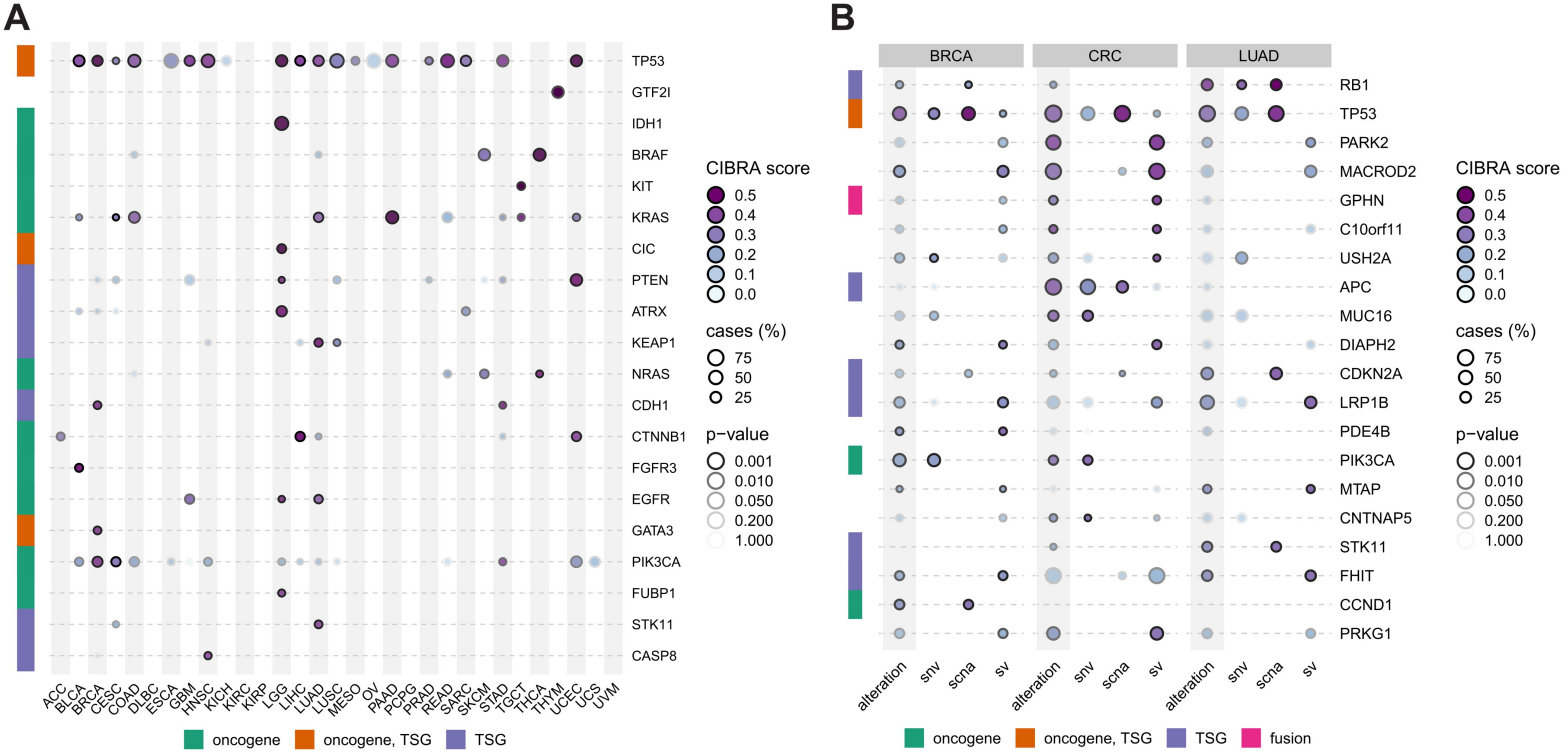
Sorted gene overview of the top 20 CIBRA impact-scoring genes from genome-wide screens. (**A**) An overview of CIBRA impact scores for genes affected by SNVs for primary cancer samples from 33 cancer types (TCGA). High-TMB samples (> 10 TMB) were excluded to create more homogeneous cohorts. The genes were sorted by impact score. (**B**) An overview of CIBRA impact scores for genes affected by genomic alterations in metastatic breast, colorectal (CRC), and lung cancer (HMF). The CIBRA score for any type of genomic alteration (highlighted in gray) and specific alterations (SNV, SCNA, and SV) are shown. Row annotations indicate the driver role of genes classified in tumor suppressor genes (TSG; purple), oncogenes (green), either (orange), and fusions (pink) retrieved from the COSMIC cancer gene census database. Dot color indicates the CIBRA impact score, the dot edge color indicates the significance of the CIBRA impact score assessed with a permutation test, and the size of the dot indicates the prevalence (%) of genomic alteration with a minimum of 10 cases.

In order to assess the ability of CIBRA to identify known tumor suppressor genes and oncogenes, the impact of coding SNVs was measured by performing genome-wide screens in 33 cancer types using data from The Cancer Genome Atlas (TCGA). First, coding SNVs retrieved from processed whole exome sequencing data (WES) were grouped at the gene level. A gene is considered to be affected in a sample if a coding SNV is present within the gene. The system-wide impact was evaluated for any protein-coding gene within the genome with at least 10 cases within the cancer type tested using transcriptomics data. Of all genes affected by coding SNVs, 8.9% (92) were identified to have a significant system-wide impact with an adjusted *P*-value ≤ 0.01. From these genes, 54.3% (50) are experimentally validated oncogenes and tumor suppressor genes registered in the COSMIC cancer gene census (CGC) (Sondka *et al.* 2018). The performance of CIBRA in identifying cancer genes evaluated with the CGC using the area under the curve of a receiver operating characteristic (ROC-AUC) is 0.79 (Supplementary Fig. S6). This is substantially higher than other recent driver detection methods using only genomics data as benchmarked in Shi *et al.* (2022). An overview of the top 20 genes ordered on the CIBRA impact score is shown in Fig. 2A. The full results of the screens are reported in Supplementary Table S1. Of the 20 top-scoring genes, most (95%) are known cancer genes. However, some genes such as General Transcription Factor IIi (*GTF2I*) are not (yet) registered as known tumor suppressor genes and oncogenes in the COSMIC cancer gene census database. Nevertheless, *GTF2I* has gained recent attention as a characteristic cancer gene associated with spindle cell morphology in thymomas (Bille *et al.* 2022, Giorgetti *et al.* 2022,). These results show that CIBRA is capable of detecting the impact of known biologically relevant genes affected by SNVs.

### 3.2 Structural variants have a significant system-wide impact in metastatic cancer

Next, we assessed the biological impact of genomic alterations, such as SCNAs and SVs, that have been difficult to assess using frequency-based detection methods. A genome-wide screen of genes affected by SNVs, SCNAs, SVs, or any of the alterations mentioned before was performed on metastasized breast, lung, and colorectal cancer (CRC) using data from the Hartwig Medical Foundation (HMF). The availability of both deep-WGS data and matched RNA-seq data allowed us to assess the impact of SNVs, SCNAs, and SVs using CIBRA. From the genome-wide screen, 16.5% (598) of genes affected by any of the three alteration types were identified to have a significant system-wide impact with an adjusted p-value ≤ 0.01 (Fig. 2B and Supplementary Table S2). SVs show to have a large effect on the system, with 30.3% (421) of genes affected by SVs having a significant system-wide impact, especially in CRC (Fig. 2B and Supplementary Table S2). However, from the list of any type of alteration with a significant system-wide impact, only 12.4% (74) are registered in the COSMIC cancer gene census. After the inclusion of SVs and SCNAs, most significant CIBRA hits are not registered as oncogenes and tumor suppressor genes (Fig. 2B). Only 8.8% (37) of the genes affected by SVs were registered. In particular, two genes located within genomic regions referred to as “common fragile sites,” Mono-ADP Ribosylhydrolase 2 (*MACROD2*) and Parkin RBR E3 Ubiquitin Protein Ligase (*PRKN*, also known as *PARK2*) are among the top 10 genes. Although common fragile sites are genomic regions prone to accumulate SVs under replicative stress, *PRKN* shows a significant CRC-specific signal. Moreover, *MACROD2* shows a significant effect in CRC and breast cancer. These results show that SVs have a significant system-wide impact in colorectal, breast, and lung cancer to a degree similar to that of known oncogenes and tumor suppressor genes.

### 3.3 Assessing gene impact by coding effect and SCNA status

**Figure 3.**
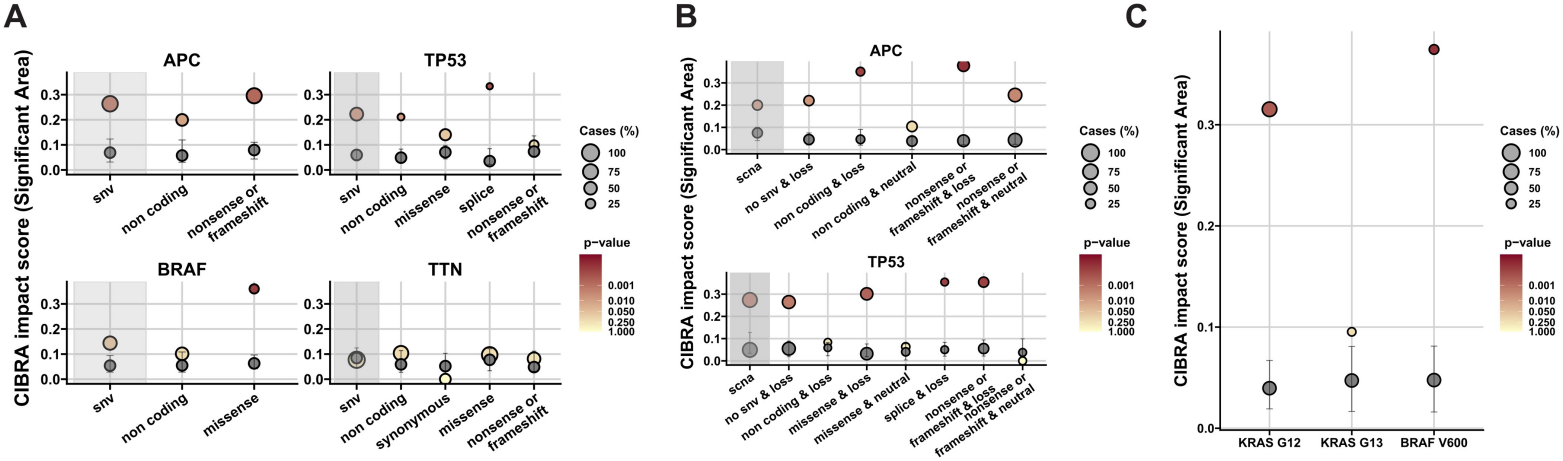
Refining genomic alterations by mutation type and genomic location for the identification of impactful alterations using CIBRA. (**A**) CIBRA impact score for SNVs dissected in their underlying coding effects: non-coding, synonymous, missense, splice, and nonsense or frameshift for four frequently mutated genes in metastatic colorectal cancer: *APC*, *TP53*, *BRAF*, and *TTN* using data from metastatic microsatellite stable CRC. Two of the genes, *APC* and *TP53*, are known tumor suppressor genes. *BRAF* is a known oncogene, and *TTN* is a recurrently mutated gene without a known cancer-associated function in CRC. (**B**) CIBRA impact score for SCNAs combined with SNV coding effects for the TSGs *APC* and *TP53* in metastatic microsatellite stable CRC. (**C**) CIBRA impact score visualization of *KRAS* codon 12 (G12), 13 (G13), and *BRAF* codon 600 (V600) mutations. (A), (B), and (C) Shaded gray area: the impact score of the overall effect of SNVs or SCNAs. Gray dot: median permutation CIBRA score, with the error bars representing the IQR. Dot color: significance of the CIBRA score assessed by a permutation test. Dot size: prevalence of the alteration (%).

Thus far, we have explored the impact of genomic alterations at the gene level. However, CIBRA can also be used to investigate if a certain type of mutation has more impact than other alterations in the same gene. Here, we explore how CIBRA can be used to identify the most impactful mutation type by systematically testing different SNVs, classified according to their coding effect, for known tumor suppressor genes (*APC* and *TP53*) and oncogenes (*BRAF*) in metastasized microsatellite stable CRC. First, the impact of SNVs classified according to their coding effect was assessed. The overall system-wide impact of SNVs can be decomposed into their underlying coding effects showing stronger and weaker signals (Fig. 3A). For example, if we consider all SNVs in *BRAF*, we obtain a non-significant CIBRA score (0.144, *P* > 0.05). However, if we focus only on missense variants, a significant high impact score can be observed (CIBRA score: 0.360, *P* ≤ 0.0001). This is consistent with the expectations of the oncogene *BRAF*, where missense variants, specifically V600E, have been reported to be oncogenic variants in colorectal cancer (Loupakis *et al.* 2009, Kopetz *et al.* 2019). On the other hand, the frequently mutated gene *TTN* shows no improvement in signal when refining the type of mutation in colorectal cancer. This aligns with the notion that due to the size of *TTN* there is an accumulation of mutations associated with the tumor mutational burden without a defined effect in colorectal cancer (Muzny *et al.* 2012, Oh *et al.* 2020). A trend similar to *BRAF* can be observed in *TP53*, where the splice variants show the highest impact score (CIBRA score: 0.334, *P* ≤ 0.0001). However, for tumor suppressor genes such as *APC* and *TP53* another refinement layer is needed. If we include SCNA status along with the SNV coding effect, the CIBRA impact score increases for both *APC* and *TP53* (Fig. 3B). A loss in conjunction with a coding SNV shows the highest CIBRA impact score. For *APC*, the highest impact score was reached with the combination of nonsense or frameshift variants and copy number loss (CIBRA score: 0.378, *P* < 0.0001), while with only nonsense or frameshift variants, the impact score was similar to the effect of only copy number loss (CIBRA score: 0.245, *P* < 0.005). A similar effect can be observed for *TP53*. This reaffirms the notion that tumor suppressor genes require two hits to have the most effect and shows that CIBRA can detect this property (Berger *et al.* 2011, Datta *et al.* 2020). Hence, we can use CIBRA to refine the coding effect by observing which type of alteration has the most impact within a gene.

### 3.4 Refining gene impact by zooming in on genomic location

The most impactful mutation within a cancer gene could be identified by refining on mutation type. However, the genomic location of an alteration can also influence the impact. To exemplify this, we explored the impact of mutations within subregions in *KRAS* and *BRAF*. If we focus on *KRAS* and dissect the coding variants on their genomic location, only codon 12 (G12) variants showed a significant system-wide impact in metastatic microsatellite stable CRC (CIBRA score: 0.32, *P* < 0.001; Fig. 3C). This is in line with the distinct clinical behavior between codon 12 and 13 (Yoon *et al.* 2014, Jones *et al.* 2017). Moreover, codon 12 variants show an impact to a similar extent as *BRAF* codon 600 variants (CIBRA score: 0.37, *P* < 0.001; Fig. 3C).

To demonstrate how the impact of SVs may change in different subregions of a gene, *MACROD2*, one of the most impactful genes affected by SVs in our CRC screen, was considered. *MACROD2* lies within a common fragile site, a genomic location prone to acquire focal deletions when the cell is under replicative stress. Given the location of *MACROD2*, the question arises whether the high impact observed from the genome-wide screen (Fig. 2B) is the effect of breakage within the fragile site due to replication stress or if specific variants within *MACROD2* are associated with the observed impact. When zooming in on SVs affecting non-coding regions, coding regions outside the MACRO domain or inside the MACRO domain, SVs affecting the MACRO domain of *MACROD2* result in the highest CIBRA impact score (Supplementary Fig. S7A). If we further zoom in on the specific exons, the impact of SVs changes depending on which exon of *MACROD2* is affected. Specifically, SVs affecting exons 5 and 6 result in the highest impact scores (Supplementary Fig. S7B). These results are in line with the work by Sakthianandeswaren *et al.* (2018) showing that the impact of *MACROD2* is not an epiphenomenon from the breakage of the common fragile site and, moreover, results in distinct clinical behavior (van den Broek *et al.* 2018). Therefore, these results show that CIBRA allowed to establish a better definition of which type of SVs in MACROD2 are impactful.

### 3.5 Distinct expressional similarity pattern between *KRAS* and *BRAF* coding variants in metastatic colorectal cancer

**Figure 4.**
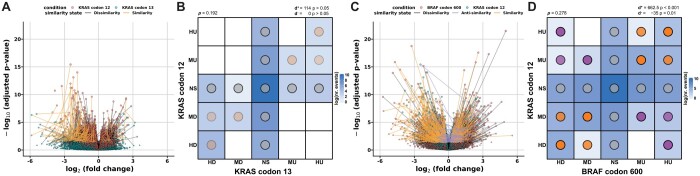
Biological similarity assessment between two genomic alterations (conditions) in metastatic microsatellite stable colorectal cancer (HMF). (**A**) Linked volcano plot showing the *log*_2_ fold change (FC) and the adjusted *P*-value of genes differentially expressed between *KRAS* G12 variants (red) and WT and *KRAS* G13 variants (blue) and WT. The similarity (orange), anti-similarity (purple), and dissimilarity (gray) states are shown by the colored links between the corresponding genes. (**B**) Categorical DE similarity pattern between *KRAS* G12 and G13 variants compared to *KRAS* WT. The color indicates the log-transformed number of events (genes) corresponding to the DE state combinations. The dot color indicates the type of similarity present in the pattern, and the intensity is proportional to the *P*-value of the similarity score with an upper bound of 0.001. The directional similarity has been reported as similarity and anti-similarity with the corresponding *P*-value assessed with a permutation test. (**C**) Linked volcano plot between *BRAF* V600 variants and *KRAS* and *BRAF* WT (red) and *KRAS* G12 variants and WT (blue). (**D**) Categorical DE similarity pattern between *KRAS* G12 and *BRAF* V600 variants compared to *KRAS* and *BRAF* WT samples.

To assess if two alterations have the same gene expression patterns, CIBRA can derive a similarity score between the distinct genomic alterations. The CIBRA similarity score compares two genomic alterations in contrast to the same control condition and quantifies the extent of similarity between the two conditions. To illustrate the similarity score, variants within the same gene and variants between different genes are compared. For the within-gene comparison, *KRAS* codon 12 and codon 13 variants are considered using data from microsatellite stable metastatic CRC. No significant similarity was observed between codons 12 and 13 (*d*^+^ = 114, *P* > 0.05; Fig. 4A and B). This difference can be mainly attributed to the lack of DE genes for codon 13 that are observed for codon 12. This highlights that codon 12 variants have a stronger distinct effect on the system. However, there are also a few significant DE genes for codon 13 that are also captured by codon 12 as shown by the light diagonal line in Fig. 4B. On the other hand, if we focus more closely on *KRAS* G12 and *BRAF* V600 variants, two genes in the same pathway with a significant system-wide impact, a distinct significant pattern of similarity (*d*^+^ = 662.5, *P* < 0.001) and anti-similarity (*d*^−^ = −35, *P* < 0.01) can be observed (Fig. 4C and D). This highlights that although both genes are part of the epidermal growth factor receptor (EGFR) pathway, they show a different effect on the system in metastatic microsatellite stable CRC. This is in line with the different clinical behaviors reported between *KRAS* and *BRAF* variants in CRC (Morkel *et al.* 2015).

### 3.6 CIBRA versus machine learning (ML)

Last, an alternative method was evaluated to determine the impact of genomic alterations. We hypothesized that if a mutation has an impact on the expression levels of the biological system, it should be possible to predict the mutation status of a sample from the genome-wide expression profile. Hence, the prediction performance of the model provides an indication of the impact of a variant. A machine learning (ML) model was trained to predict the mutation status of five known cancer genes given transcriptomics data. The performance scores for these models were subsequently compared to the CIBRA impact score for five known cancer genes in metastatic CRC: *TP53*, *APC*, *KRAS*, *BRAF*, and *PIK3CA*, as well as a gene often mutated due to its size, *TTN*, with no reported impact. The model performance to predict the mutation status of the six assessed genes is shown in Supplementary Table S3. Various definitions of mutations have been evaluated, including the combination of SNV and SCNA in the tumor suppressor genes *TP53* and *APC*. The mutation status of the cancer genes is indeed predictable from their expression profiles as shown by ROC-AUC scores (Supplementary Table S3). However, not all mutation definitions could be assessed using the ML approach, as the ML model needed at least 50 cases and controls to be reasonably trained and validated. In contrast, the CIBRA impact score can be calculated for all genomic alterations in Supplementary Table S3, and could calculate the impact score with a minimum of 10 cases and controls (Supplementary Fig. S2). Moreover, CIBRA was able to identify the system-wide impact of all cancer genes evaluated and confirms that TTN does not have a system-wide impact in CRC, as also observed with the ML model. Overall, these results show that both the performance scores of the ML model and the CIBRA impact score can be used as impact indicators for genomic alterations, while the CIBRA impact score is preferred when a small number of samples are available for a specific alteration.

## 4 Discussion

Identifying alterations with a system-wide impact is a challenging task. CIBRA integrates two different omics data types to determine the system-wide impact of genomic alterations. By integrating multi-omics data, CIBRA was able to identify the majority of known tumor suppressor genes and oncogenes from a pan-cancer genome-wide screen of primary cancers. Notably, when we applied CIBRA on a genome-wide screen of genes affected by SNVs, SCNAs, and SVs using data from metastatic colorectal, breast, and lung cancer, many genes affected by SVs showed a significant system-wide impact with a similarly high impact as known cancer genes such as *TP53* and *APC*. This finding suggests that the impact of structural variants has been largely underestimated. Furthermore, CIBRA could systematically refine genomic alterations on genomic sublocations and mutation types to narrow impactful mutations. Finally, CIBRA could facilitate the evaluation of similarities between genomic alterations in terms of their impact. The systematic identification of the most impactful type of mutation and gene subregion can facilitate the prioritization and design of focused experimental assays to validate the observed system-wide impact and allow for the reassessment of their clinical implications. In conclusion, CIBRA is a versatile method for identifying genomic alterations with a system-wide impact on tumor biology and has the potential to help in our understanding of disease-associated genomic alterations.

In this work, we have shown that the impact of genomic alterations can be determined in two ways by integrating multi-omics data: a statistical method that determines the system-wide impact of genomic alterations, i.e. CIBRA, and a machine learning model that identifies characteristic changes in the system associated with the genomic alteration. Machine learning methods such as a random forest model have the strength that they can identify non-linear relationships that do not necessarily consist of system-wide effects, allowing them to identify a wider range of cancer gene effects. However, these methods require sufficient data to train and validate a model, limiting their scope of use. In contrast, CIBRA is able to determine the system-wide impact of any genomic alteration with at least 10 cases and controls, allowing us to explore a wider and deeper space, given the assumption that the genomic alteration has its impact in a linear system-wide effect.

From the pan-cancer genome-wide screen of primary cancers, 67% of known tumor suppressor genes and oncogenes affected by any coding SNV showed a significant system-wide change in the transcriptome. We hypothesized that biologically relevant alterations elicit large changes throughout the system. As hallmarks of cancer are central components of the cell and the tumor, disruption of these processes can induce large trickle-down effects through the system, explaining the many known cancer genes found with a large system-wide effect (Hanahan 2022). Although cancer genes are often key components of hallmarks of cancer, genomic alterations within these genes do not necessarily necessitate a large change in the system (Sanchez-Vega *et al.* 2018). Even though we have used a broad definition, i.e. any SNV in the gene, CIBRA was able to detect these trickle-down effects from cancer genes and verified our hypothesis that cancer genes can elicit such large effects through the system. Notably, not all of the highest-scoring genes are registered tumor suppressor genes and oncogenes, such as *GTF2I*. However, even though they are not registered in the cosmic cancer gene census, recent studies have shown the tumorigenic potential of *GTF2I* in thymoma (Bille *et al.* 2022, Giorgetti *et al.* 2022). On the other hand, from our metastatic breast, colorectal, and lung cancer screens, SVs emerged as having a significant system-wide impact on cancer. Notably, genes within common fragile sites, such as *PRKN* and *MACROD2*, which are among the top 10 scoring genes, showed an exorbitant impact in metastasized CRC, highlighting their potential relevance in cancer. *MACROD2* has been reported to promote chromosomal instability, positioning it as a potential tumor suppressor gene (Sakthianandeswaren *et al.* 2018). Variants within *PRKN* have been reported to result in mitotic instability, contributing to oncogenesis (Veeriah *et al.* 2010). Our findings suggest that the impact of SVs in cancer has thus far been under-reported, and more attention is needed for SVs in the field of cancer biology and precision oncology.

Systematically refining the definition of alterations on mutation type and genomic location can be helpful in two aspects: first, to prioritize and design focused experiments for validating the impact, and second, to select potential biomarkers for clinical testing and validation. Due to the limited size of patient cohorts, clinical data is often insufficient to fully explore the potential biomarker space (Ren *et al.* 2020). CIBRA enables the prioritization of biomarkers by pre-selecting and refining their definition. In this way, noisy definitions can be refined to allow for more effective testing and validation of clinical markers with limited clinical data.

There are also some limitations to the CIBRA methodology. First, the presence or absence of impact is not an indication of a causal effect. It only represents a potential association with changes in the biology of the system. Moreover, the homogeneity of the set of samples influences the power of the CIBRA impact and similarity scores. The more homogeneous the set of samples, the better the signal-to-noise ratio becomes. Given the absence of a gold-standard dataset for benchmarking cancer driver detection methods, along with our use of a broad definition in the genome-wide screens, we anticipate that the reported statistics are an underestimation of the potential performance of CIBRA. However, through refinement, we can improve the performance as shown by the examples in Fig. 3. Despite these challenges, CIBRA was able to identify known and novel cancer genes. In addition, both the systematic refinement of genomic alterations and the similarity score are powerful tools to help improve our understanding of cancer biology.

## Supplementary Material

btae384_Supplementary_Data

## Data Availability

All datasets analyzed in this study are publicly available. The data generated in this study are available within this article, its supplementary data files, and the code and data repositories.
