# Supplementary material for: CIBRA identifies genomic alterations with a system-wide impact on tumor biology
Source: Bioinformatics. 2024 Sep 4;40(Suppl 2):ii37–44. doi: 10.1093/bioinformatics/btae384 (PMC11373315; doi:10.1093/bioinformatics/btae384)
Supplement: btae384_Supplementary_Data [file btae384_supplementary_data.zip › supplemental_figure_2.pdf]

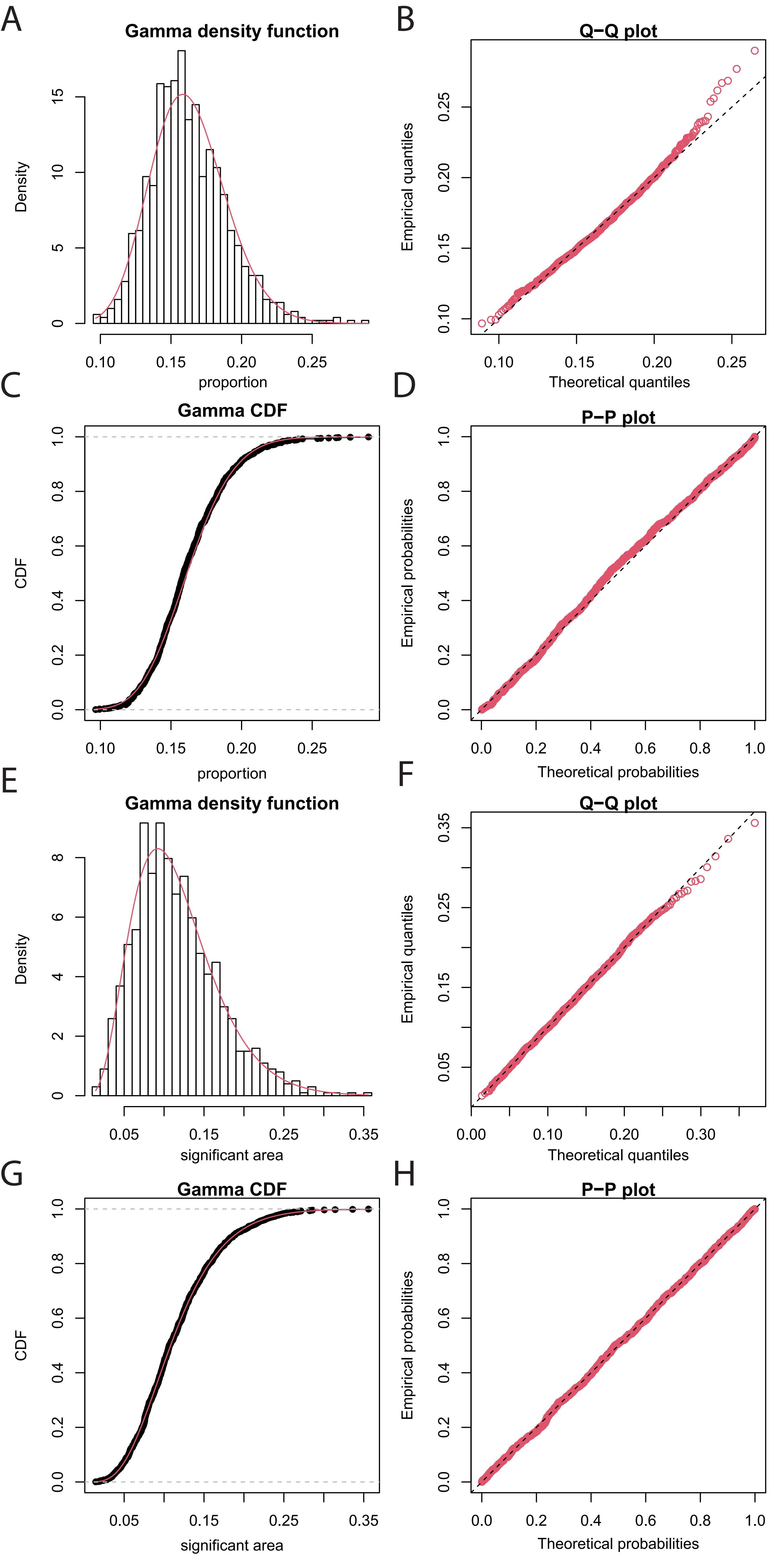

A
Gamma density function
Density
proportion
B
Q−Q plot
Empirical quantiles
Theoretical quantiles
C
Gamma CDF
CDF
proportion
D
P−P plot
Empirical probabilities
Theoretical probabilities
E
Gamma density function
Density
significant area
F
Q−Q plot
Empirical quantiles
Theoretical quantiles
G
Gamma CDF
CDF
significant area
H
P−P plot
Empirical probabilities
Theoretical probabilities
