# Supplementary material for: CIBRA identifies genomic alterations with a system-wide impact on tumor biology
Source: Bioinformatics. 2024 Sep 4;40(Suppl 2):ii37–44. doi: 10.1093/bioinformatics/btae384 (PMC11373315; doi:10.1093/bioinformatics/btae384)
Supplement: btae384_Supplementary_Data [file btae384_supplementary_data.zip › supplemental_materials.pdf]

1. Supplemental materials and methods

To identify genomic alterations with a system-wide impact, we developed a computational method called Computational Identification of Biologically Relevant Alterations (CIBRA). We hypothesized that biologically relevant alterations elicit specific changes through the system that reflect the genomic change, whereas alterations without an impact have no consistent systemic change on the system. As such, by assessing the degree of change in the system, the extent of the impact of a genomic alteration can be determined.

1.1. CIBRA scores

CIBRA probes system-wide responses based on samples with (cases) and without (controls) genomic alterations using a Beta-Uniform mixture model (Pounds and Morris, 2003) to decompose the p-value distribution generated from differential expression (DE) analysis. CIBRA has two scores, the CIBRA impact score (Fig. 1) and the similarity score (Fig. S1). For the CIBRA impact score, two measures are calculated: the significant area between the Beta and Uniform component of the Beta-Uniform mixture model and the proportion of p-values below a given p-value threshold τ. The statistical significance of the impact measures is assessed by performing 1000 sample permutations and assessing the observed impact score with respect to the permutation distribution (Fig. 1). For the CIBRA similarity score, group definitions with shared controls are used to perform differential expression analysis. The generated log_2 fold changes and adjusted p-values are used to define differential expression (DE) states. These states assign genes in whether they are significantly up- or down-regulated or if they are not significantly changed. From the list of DE states, a similarity and anti-similarity score is calculated between the conditions (Fig. S1). The significance of the scores is assessed with a permutation test.

1.1.1. Differential expression analysis

To assess the system-wide response of biological alterations, first, a differential expression (DE) analysis was performed. Any DE analysis method that outputs a valid p-value distribution for omics data, i.e. a p-value distribution that is uniform under the null hypothesis can be used with CIBRA. In this work, DESeq2 (version 1.38.2, default parameters, RRID:SCR_015687 (Love et al., 2014)), edgeR (version 3.40.1, default parameters, RRID:SCR_012802 (Robinson et al., 2010)) and Limma-Voom (version 3.54.0, default parameters, RRID:SCR_010943) were assessed. The results reported within this work have been generated using DESeq2, as we have observed that it tends to mostly give valid p-value distributions and is capable of being executed in parallel. EdgeR tended to also mostly show a valid p-value distribution. Limma-Voom gave more invalid p-value distributions with an inflation of 1 using our data compared to the other two methods. Zero variance and low count genes (<10 total counts) were excluded from the analysis.

1.1.2. Beta-Uniform mixture model

To estimate the system-wide effect size of a genomic alteration, the p-value distribution derived from DE analysis was decomposed using a Beta-Uniform mixture model (Pounds and Morris, 2003). The model is a composite of the Beta distribution with parameter $\beta = 1$ and a uniform distribution. The mixture model has a probability density function (pdf), which can be calculated as:

$$f(x \mid \alpha, \lambda) = \lambda + (1-\lambda)\alpha x^{(\alpha-1)} \quad for\ x, \lambda, \alpha \in (0,1) \qquad (1)$$

where the probability of x is dependent on the shape parameters λ and α for $x, \lambda, \alpha \in (0,1)$. The Beta-Uniform mixture model was fitted using the R package BioNet (version 1.58.0, (Beisser et al., 2010)).

For the Beta-Uniform mixture model, under the null hypothesis that the genomic alteration has no system-wide expression change, the model will lead to a Uniform function with $\lambda = 1$. Under the alternative hypothesis that the genomic alteration has a system-wide expression change, the function will acquire density for small p-values and $0 \leq \lambda < 1$. The larger the signal, the larger the density the function will acquire. Given these hypotheses, we have derived two signal measures that capture the system-wide expression change: the CIBRA impact score termed the *significant area* and the *proportion*.

CIBRA impact score (significant area): The significant area is the integral between the Beta and Uniform components calculated as:

$$\int_0^1 f(x)dx = \alpha(1-\lambda)(x^{\alpha-1}-1) \quad for\ \lambda, \alpha \in (0,1) \qquad (2)$$

where λ and α are the estimated shape parameters of the Beta-Uniform mixture model. The significant area indicates the extent of p-values that arise from the alternative component, indicating the extent of change in the system. Under the null hypothesis of no system-wide change, the significant area is expected to be 0, while it increases with an increasing change in the system up to a theoretical maximum of 1 (Fig. 1A).

CIBRA impact score (proportion): the proportion of p-values smaller than a significant threshold value (τ). In this study, the threshold τ was set to 0.1. The proportion has been taken as a signal measure to accommodate and detect biases in the p-value distribution. If the p-value distribution shows a shift in values toward 1, given the characteristics of p-values, the distribution is deemed invalid. The proportion reflects this bias, as a shift in p-values toward 1 results in fewer p-values below τ, because if there is no signal in the data, a p-value distribution tends to behave uniform. As such, the proportion should be τ under the null hypothesis, and if below τ, is an indication of an invalid p-value distribution. The proportion can range from 0 to 1.

The significant area is the measure that describes the extent of the system-wide impact and will be referred to as the CIBRA impact score in this manuscript, while the proportion can give an indication of the extent of significant changes in the system. In addition, the proportion can be used to determine whether the observed values are valid.

1.1.3. Permutation test

To estimate the significance of the CIBRA impact scores given the variation present in the dataset, a permutation test was performed. For all data platforms (TCGA and HMF) and cancer types, at least 1000 sample permutations were made with a case-control parameter grid with 30 steps for the case size and 5 steps for the control size, both starting at 10 and increasing up to the maximum number of samples. The overall distributions for the CIBRA impact scores were found to be well-fitted by a gamma distribution, as shown in Fig. S3. The cancer type and data platform are confounding factors shifting the distribution (Fig.

S4; $p < 0.0001$) when taken in a Gamma regression model fitted using a generalized linear model (GLM) with family Gamma and an inverse link function. As such, the permutation distribution must be generated for each cohort (i.e., for each cancer type). The influence of the number of cases and controls on the characteristics of the permutation distribution was also assessed. Given that the case and control sizes are general properties shared between cancer types and data platforms, the influence of case and control sizes has been assessed on one data set. A GLM with family Gamma and an inverse link function was fitted on the CIBRA impact scores calculated from metastatic colorectal cancer data (HMF). No significant relationship was found between the number of cases and controls and the CIBRA impact scores. As such, a generic permutation distribution could be made for each cohort. However, a low number of cases and controls does result in more invalid CIBRA impact scores, i.e., a proportion below τ, meaning an invalid p-value distribution, as shown in Fig. S2. Moreover, for less than 8 cases/controls, DESeq2 failed to perform DE analysis on our data. As such, we recommend at least 10 cases/control to have reliable results. To estimate the significance of the signal measures, the signal measures are compared to a Gamma distribution fitted against the corresponding 1000 permutations of the cancer type and data platform. The fit was estimated using the R package fitdistrplus (version 1.1-8, parameters: distr="gamma", RRID:SCR_024274 (Delignette-Muller and Dutang, 2015)).

1.2. CIBRA similarity score

To assess the similarity in system-wide expression impact between two alterations, e.g., two different variants within the same gene, we derived a similarity score. The similarity score uses the adjusted p-values (p) and log_2 fold changes (FC) generated from DE analysis to estimate the distance between two alterations within this space. For DE analysis, shared controls are needed as the similarity score assumes a shared reference point as shown in the flow chart depicted in Fig. S5. To calculate the CIBRA similarity score, first, the p-value/fold change space was divided into 5 regions: highly upregulated (HU), moderately upregulated (MU), highly downregulated (HD), moderately downregulated (MD) and not significant (NS) (Fig. S5). Genes within this space are assigned DE states (DE_s) using the boundaries in equation 3. The equation assigns a score based on the coordinates of the gene within the fold change (FC) and adjusted p-value (p) space for the given condition. Following this step, a vector of DE_s is generated for each condition. The boundaries that divide the regions are common demarcations for volcano plots in RNA-Seq data analysis to identify substantially changed genes.

$$DE_s(p, FC) = \begin{cases} HU & \text{if } FC \geq 1 \text{ and } p \leq 0.05 \\ MU & \text{else if } 0 < FC < 1 \text{ and } p \leq 0.05 \\ NS & \text{else if } p > 0 \\ MD & \text{else if } 0 > FC > -1 \text{ and } p \leq 0.05 \\ HD & \text{else if } FC \leq -1 \text{ and } p \leq 0.05 \end{cases} \tag{3}$$

To calculate the similarity between the two conditions, first, the frequency of DE_s combinations between the two conditions is calculated. This contingency table is a 5x5 matrix termed N. A weight matrix representing the similarity (D^+) and anti-similarity (D^-) relationships between the DE_s is multiplied with the contingency table (N) to calculate the directional similarity scores d^+ and d^- as described with equations 4 and 5 and shown in Fig. S5.

$$d^+ = \sum_{i=1}^{s} \sum_{j=1}^{s} D_{ij}^+ N_{ij} \tag{4}$$

$$d^- = \sum_{i=1}^{s} \sum_{j=1}^{s} D_{ij}^- N_{ij} \tag{5}$$

To assess the significance of the similarity between the two conditions, a permutation test was performed. A total of 10000 condition permutations were calculated to generate a positive and negative directional similarity score distribution. Random gene definitions from genome-wide screens were taken as conditions for permutations with shared controls. To assess the correlation between the DE_s of the two conditions, the Spearman correlation measure was calculated to assess the correlation between the two DE_s vectors. Given that the DE_s are discrete, a Spearman correlation measure was deemed more suitable.

1.3. Machine Learning

To assess if the impact of a genomic alteration could also be investigated by a machine learning model, we trained a random forest model that predicts the genomic alteration status from transcriptomics data. If the transcriptomics data contain an expression signal associated with the alteration, it should be possible to train such a machine-learning model with a reasonable performance. In this work, transcriptomics data has been used as a measure of the system changes.

1.3.1. RNA-seq processing

RNA-seq counts were pre-processed by removing zero variance genes and mapping ENSEMBL identifiers to HUGO gene symbols. Transcripts without HUGO gene symbol annotations were removed. The highest expressed transcript per gene was retained as the gene count. The count data were normalized using the TMM normalization method from the R package edgeR and transformed to scaled log counts per million (logCPM) values.

1.3.2. Random forest model

A Random Forest classifier was built using the Classification and Regression Training R package caret (version 6.0.93, RRID:SCR_021138 (Kuhn et al., 2021)). The model was trained using a 70%/30% train-test split with a 10x repeated 5-fold cross-validation (CV) loop on the training set for feature selection and parameter tuning. Recursive feature elimination (RFE) was performed with a 10-300 feature range with 25 steps. The area under the precision-recall curve (PRCAUC) calculated with the R package PRROC (version 1.3.1, (Grau et al., 2015)) was used as a performance metric. Parameter tuning was performed using the internal tuning step of the caret train function with a 10-value vector for the parameter 'mtry'. The final model performance was evaluated with the 30% test set using the area under the receiver operating characteristic curve (ROCAUC) and the PRCAUC as performance measures.

1.3.3. Permutation testing

To assess if the performance of the ML model was significantly better than random, a permutation test was performed with 100 class label permutations. The performance of the original model was compared to the permutation performance distribution. A

p-value was calculated as the fraction of permutations with a performance higher than or equal to the original model.

1.4. Data

1.4.1. The Cancer Genome Atlas

Public data from The Cancer Genome Atlas (TCGA) were gathered from the Genome Data Commons (GDC) portal for 33 cancer types (Grossman et al., 2016). Available processed single nucleotide variant calls using whole-exome sequencing data were retrieved using the R package TCGAbiolinks (version 2.25.3, (Colaprico et al., 2016)), with query parameters: data.category = "Simple Nucleotide Variation", data.type = "Masked Somatic Mutation", legacy = FALSE, access = "open" and workflow.type = "Aliquot Ensemble Somatic Variant Merging and Masking". Available RNA-Seq data were retrieved with the query parameters: data.category = "Transcriptome Profiling", data.type = "Gene Expression Quantification" and workflow.type = "STAR – Counts". Clinical data and tumor mutational burden (TMB) were retrieved from cBioPortal (Cerami et al., 2012; Gao et al., 2013; Weinstein et al., 2013). 'Silent' variants indicated by the variant classification provided in the Mutation Annotation Format (MAF) file were removed from the genome-wide screen analysis.

1.4.2. The Hartwig Medical Foundation

From the Hartwig Medical Foundation (HMF), whole genome sequencing (WGS) data was retrieved from 610 metastatic colorectal cancer samples, 996 metastatic breast cancer samples, and 551 metastatic lung cancer samples. RNA sequencing data was available from 394 metastatic colorectal cancer samples, 332 metastatic breast cancer samples, and 127 metastatic lung cancer samples. WGS data was processed with the PURPLE-GRIDDS-LINX pipeline from HMF as previously described (Priestley et al., 2019) to generate SNV, SV, and SCNA calls. RNA sequencing data were analyzed with Isofox (version 1.5, Isofox GitHub) to generate transcript counts. 'Silent' variants indicated by the SnpEff (version 4.3, RRID:SCR_005191) canonical transcript summary were removed from the SNV calls in all further analyses that indicated coding variants. SV calls were only retained when they passed all filters.

1.5. CIBRA genome-wide screen: application to identify the impact of known and novel genomic alterations

To evaluate the capability of CIBRA to detect known and novel genomic alterations with a system-wide impact, a gene-level genome-wide screen was performed using data from the TCGA and HMF. Cancer driver annotations were obtained from the COSMIC Cancer Gene Census (CGC) database (Sondka et al., 2018). Only Tier 1 genes were used for annotations. Multiple testing correction was performed using the Benjamini-Hochberg correction (Benjamini and Hochberg, 1995). To evaluate the capability of CIBRA to identify known genomic alterations with impact, the CIBRA impact score was assessed with the CGC database. Only genes following the minimal incidence criteria (10 cases) were taken in this evaluation. Genes with fewer cases could not be tested with the CIBRA method and were as such not evaluated. A Receiver Operator Characteristic (ROC) curve was constructed from the tested genes with a CIBRA impact score and the list of known drivers. Given the absence of a gold-standard dataset for benchmarking cancer driver detection methods, along with our use of a broad definition in the genome-wide screens, we anticipate that the reported statistics are an underestimation of the potential performance of CIBRA. However, through refinement, we can imporve the performance as shown by the examples in Fig. 3.

HMF: For the HMF data consisting of breast, lung, and colorectal cancer, gene alteration definitions were constructed on four levels: SNVs, SVs, SCNAs, and any of the aforementioned alterations. For the definition, any alteration, a binary labeling was constructed where if either an SNV, SV, or SCNA occurs in the gene, the gene is given the state '1'. If none of the alterations occur within the gene, the gene is given the state '0'. The same labeling was performed for the SNV, SV, and SCNA definitions. However, the definition is only constrained to the occurrence of the given alteration within the gene. Gene annotations were retrieved using the R package AnnotationHub (version 3.6.0, RRID:SCR_024227 (Morgan and Shepherd, 2023)) with the query: "AH10684".

TCGA: For the TCGA data on a pan-cancer level, gene alteration definitions were only constructed for coding SNVs. Variants classified as 'silent' were excluded from the definitions. Binary labeling was performed where genes affected by SNVs were labeled as '1' and '0' otherwise. To reduce heterogeneity within cohorts, samples with a tumor mutational burden (TMB) > 10, termed 'high' TMB samples, were excluded from the analyses. Gene annotations were retrieved using the R package AnnotationHub with the query: "AH98495"

1.6. Refining genomic alterations on mutation type and genomic location using CIBRA

While we can define genomic alterations at the gene level, we can also zoom in and make the alterations more concrete. In this work, two ways to refine genomic alterations were assessed:

Mutation type: For mutation type, the effect of SNVs classified according to their coding effects: non-coding, synonymous, missense, splice and nonsense or frameshift, SVs defined in deletions, duplications, insertions, inversions and translocations, and SCNAs in gains and losses as depicted have been assessed. The nuanced effects of mutation types have been assessed in the genes APC, TP53, KRAS, BRAF, PIK3CA, and TTN using data from microsatellite-stable CRC (HMF).

Genomic Location: For the location of alterations, 4 levels have been defined: coding regions, exons, domains, and amino acid positions. The location annotations were retrieved using the R package AnnotationHub. For domain annotations, the R packages EnsDb.Hsapiens.v75 (version 2.99.0) and EnsDb.Hsapiens.v86 (version 2.99.0) were used. A use case of the sublocation was assessed on the gene *KRAS* and *MACROD2* using data from metastatic microsatellite stable CRC (HMF).

1.7. CIBRA similarity score: application to assess the similarity of biologically relevant alterations

To assess the similarity in system-wide expression change between two conditions, a similarity score was calculated as described in section 1.2. The similarity score was calculated for two use cases using data from metastatic microsatellite-stable CRC (HMF): KRAS codon 12 compared to codon 13 variants and KRAS codon 12 compared to BRAF codon 600 variants. For the two use cases, the shared control was wild-type for both conditions. The CIBRA impact score was calculated for each condition of the two use cases as described in section 1.1. The generated p-values and

fold changes were used to calculate the CIBRA similarity score as described in section 1.2.

References

D. Beisser et al. BioNet: an R-Package for the functional analysis of biological networks. *Bioinformatics*, 26(8):1129–1130, Apr. 2010.

Y. Benjamini and Y. Hochberg. Controlling the false discovery rate: a practical and powerful approach to multiple testing. *Journal of the Royal Statistical Society Series*, 57(1):289–300, 1995.

E. Cerami et al. The cBio Cancer Genomics Portal: An Open Platform for Exploring Multidimensional Cancer Genomics Data. *Cancer Discovery*, 2(5):401–404, May 2012.

A. Colaprico et al. TCGAbiolinks: an R/Bioconductor package for integrative analysis of TCGA data. *Nucleic Acids Research*, 44(8): e71, May 2016.

M. L. Delignette-Muller and C. Dutang. fitdistrplus: An R Package for Fitting Distributions. *Journal of Statistical Software*, 64:1–34, Mar. 2015.

J. Gao et al. Integrative analysis of complex cancer genomics and clinical profiles using the cBioPortal. *Science Signaling*, 6(269):pl1, Apr. 2013.

J. Grau, I. Grosse, and J. Keilwagen. PRROC: computing and visualizing precision-recall and receiver operating characteristic curves in R. *Bioinformatics*, 31(15):2595–2597, Aug. 2015.

R. L. Grossman et al. Toward a Shared Vision for Cancer Genomic Data. *The New England Journal of Medicine*, 375(12):1109–1112, Sept. 2016.

N. Kuhn et al. Mutation-specific effects of NRAS oncogenes in colorectal cancer cells. *Advances in Biological Regulation*, 79: 100778, Jan. 2021.

M. I. Love, W. Huber, and S. Anders. Moderated estimation of fold change and dispersion for RNA-seq data with DESeq2. *Genome biology*, 15(12):550, Dec. 2014.

M. Morgan and L. Shepherd. *AnnotationHub: Client to access AnnotationHub resources*, 2023. R package version 3.8.0.

S. Pounds and S. W. Morris. Estimating the occurrence of false positives and false negatives in microarray studies by approximating and partitioning the empirical distribution of p-values. *Bioinformatics (Oxford, England)*, 19(10):1236–1242, July 2003.

P. Priestley et al. Pan-cancer whole-genome analyses of metastatic solid tumours. *Nature*, 575(7781):210–216, Nov. 2019.

M. D. Robinson, D. J. McCarthy, and G. K. Smyth. edgeR: a Bioconductor package for differential expression analysis of digital gene expression data. *Bioinformatics*, 26(1):139–140, Jan. 2010.

Z. Sondka et al. The COSMIC Cancer Gene Census: describing genetic dysfunction across all human cancers. *Nature Reviews Cancer*, 18 (11):696–705, Nov. 2018.

J. N. Weinstein et al. The Cancer Genome Atlas Pan-Cancer analysis project. *Nature Genetics*, 45(10):1113–1120, Oct. 2013.

2. Supplemental Conflict of interest

R.J.A.F. reports grants and non-financial support from Personal Genome Diagnostics, non-financial support from Delfi Diagnostics, grants from MERCK BV, grants and non-financial support from Cergentis BV, outside the submitted work; In addition, R.J.A.F. has several patents pending. S.A. reports grants and non-financial support from Cergentis BV, Olink, Quanterix, and a patent pending, outside the submitted work. S.L. reports non-financial support from Cergentis BV and a patent pending, outside the submitted work. J.H. reports a patent pending, outside the submitted work. G.A.M. is co-founder and board member (CSO) of CRCbioscreen BV, CSO of Health-RI (Dutch National Health Data Infrastructure for Research & innovation), and member of the supervisory board of IKNL (Netherlands Comprehensive Cancer Organisation). G.A.M. non-financial support from Exact Sciences, non-financial support from Sysmex, non-financial support from Sentinel CH. SpA, non-financial support from Personal Genome Diagnostics (PGDX), non-financial support from DELFI, other from Hartwig Medical Foundation, grants from CZ (OWM Centrale Zorgverzekeraars groep Zorgverzekeraar u.a), other from Royal Philips, other from GlaxoSmithKline, other from Keosys SARL, other from Open Clinica LLC, other from Roche Diagnostics Nederland BV, other from The Hyve BV, other from Open Text, other from SURFSara BV, other from Vancis BV, other from CSC Computer Sciences BV, outside the submitted work; In addition, G.A.M. has several patents pending. The other authors declare no potential conflicts of interest.

3. supplemental figures

Table S1. Full results of the genome-wide pan-cancer screen of genes affected by SNVs using data from the cancer genome atlas. Only low TMB (< 10) samples were taken along in the screen.

Table S2. Full results of the genome-wide screen of genes affected by SNVs, SCNAs, SV, and any alterations in metastatic breast, colorectal, and lung cancer.

Table S3. Comparing the CIBRA score and machine learning models to identify the impact of genomic alterations using transcriptomics data. Both the CIBRA impact score and a random forest (RF) classification model performance score were used to assess the impact of coding SNVs, SCNAs, the combination (SNV + SCNA), or any alteration in *TP53*, *APC*, *KRAS*, *BRAF*, *PIK3CA*, and *TTN*. The impact is measured with the significant area for CIBRA and the receiver operating characteristic area under the curve (ROC AUC) for the RF model. Significance is determined through a permutation test for both methods.

			CIBRA		Machine Learning (RF)	
Gene	Alteration	Cases (samples)	Significant area (score)	p-value	ROCAUC (score)	p-value
TP53	only SNV	35	0.094	0.38	-	-
	only SCNA	133	0.298	0.007	0.92	≤ 0.01
	SNV & SCNA	144	0.378	0.001	0.94	≤ 0.01
	any alteration	327	0.327	0.004	0.95	≤ 0.01
APC	only SNV	173	0.306	0.006	0.86	≤ 0.01
	only SCNA	48	0.329	0.004	-	-
	SNV & SCNA	67	0.383	0.001	0.88	≤ 0.01
	any alteration	303	0.357	0.003	0.80	≤ 0.01
KRAS	only SNV	163	0.210	0.05	0.84	≤ 0.01
	any alteration	165	0.212	0.04	0.81	≤ 0.01
BRAF	only SNV	51	0.280	0.01	0.74	≤ 0.01
	any alteration	61	0.214	0.04	0.75	≤ 0.01
PIK3CA	only SNV	60	0.325	0.004	0.61	0.16
	any alteration	61	0.319	0.004	0.66	0.01
TTN	only SNV	210	0	1	0.52	0.38
	any alteration	219	0	1	0.4	0.93

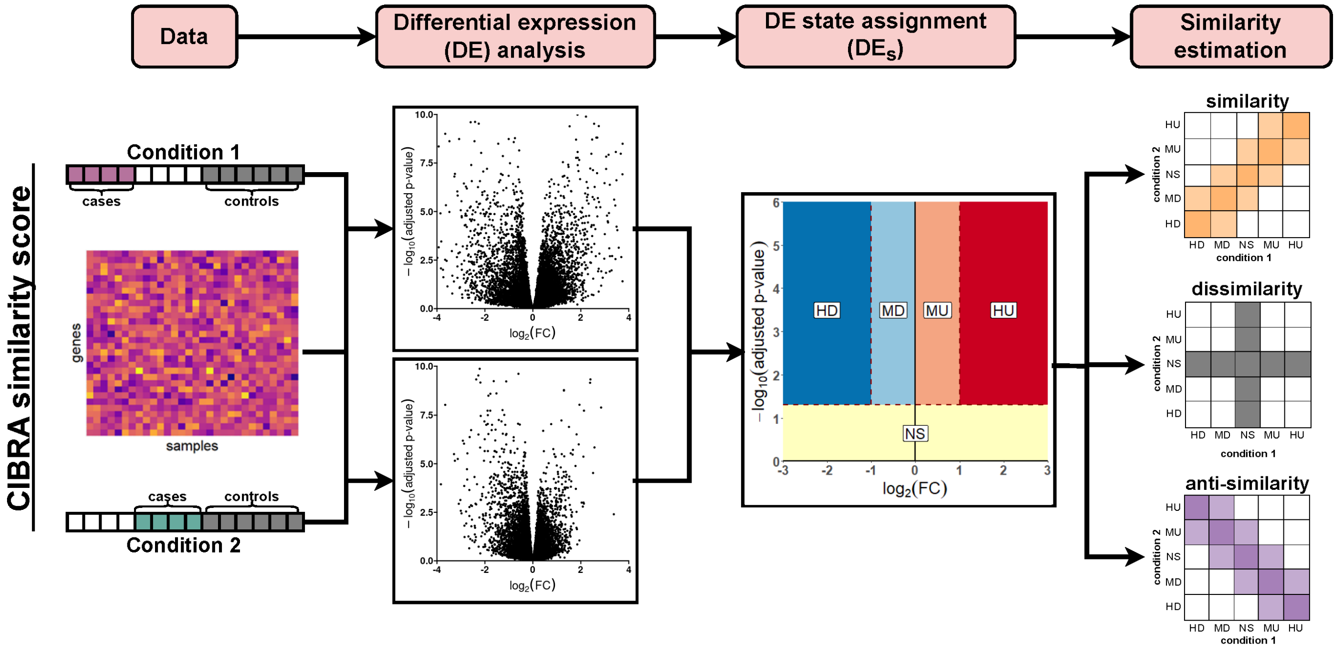

Fig. S1: Flowchart of the CIBRA similarity score calculation. Gene expression data and group definitions with shared controls were used to perform differential expression (DE) analysis. Using the generated log_2 fold changes (FC) and adjusted p-values, five DE states (DE_s): highly upregulated (HU), moderately upregulated (MU), moderately downregulated (MD), highly downregulated (HD) and not significant (NS) were assigned per condition for each gene given the corresponding p-value and fold change. With the vectors of DE_s, a similarity and anti-similarity score is calculated between the conditions and visualized using a similarity matrix. A permutation test is performed to estimate the significance of the similarity and anti-similarity scores.

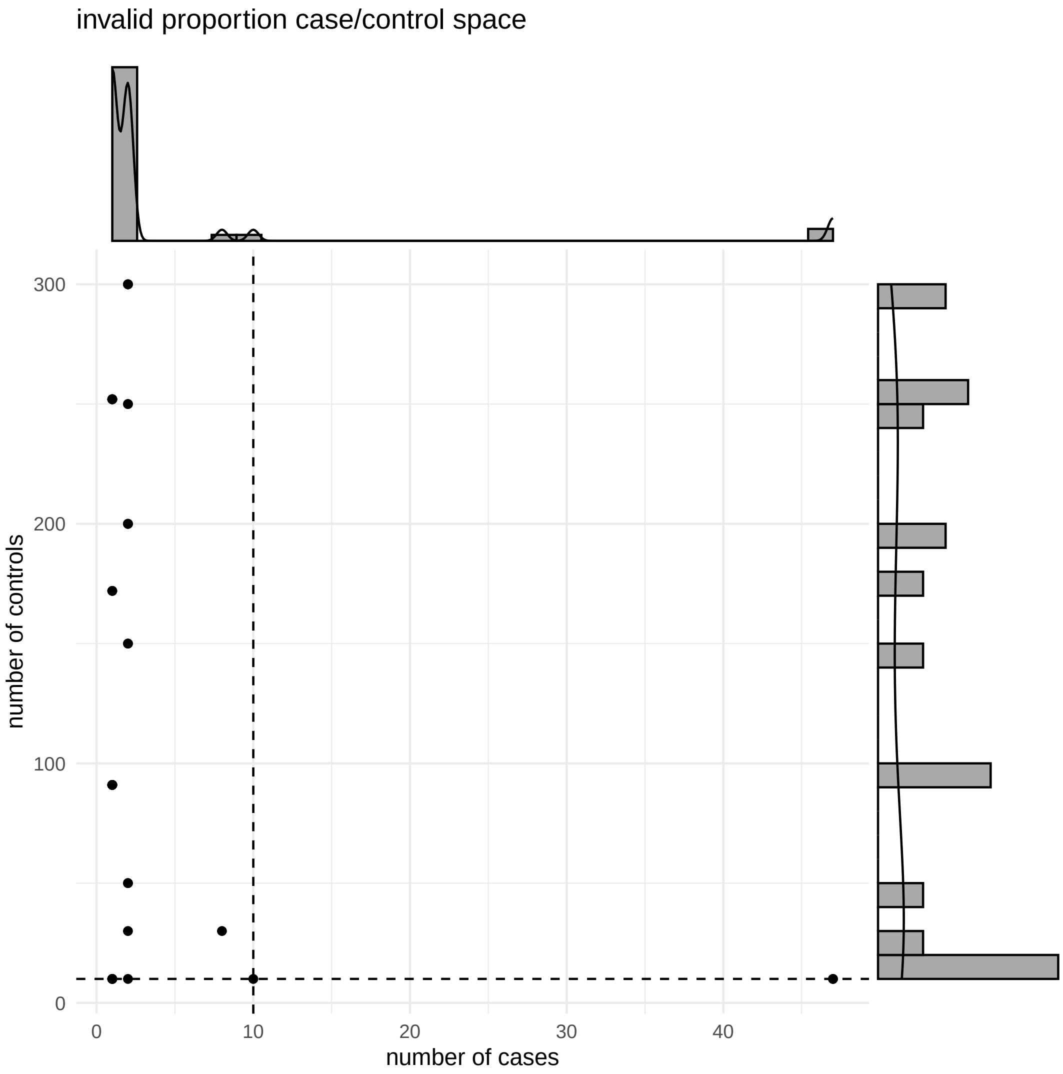

Fig. S2: Number of cases plotted against the number of controls that resulted in an invalid proportion below the threshold τ assessed with 1000 sample permutations. The threshold τ used in this manuscript is 0.1. Sample permutations with fewer than 10 cases resulted in invalid signal measures. The number of cases between 3 and 8 could not be assessed with DESeq2, as they resulted in errors.

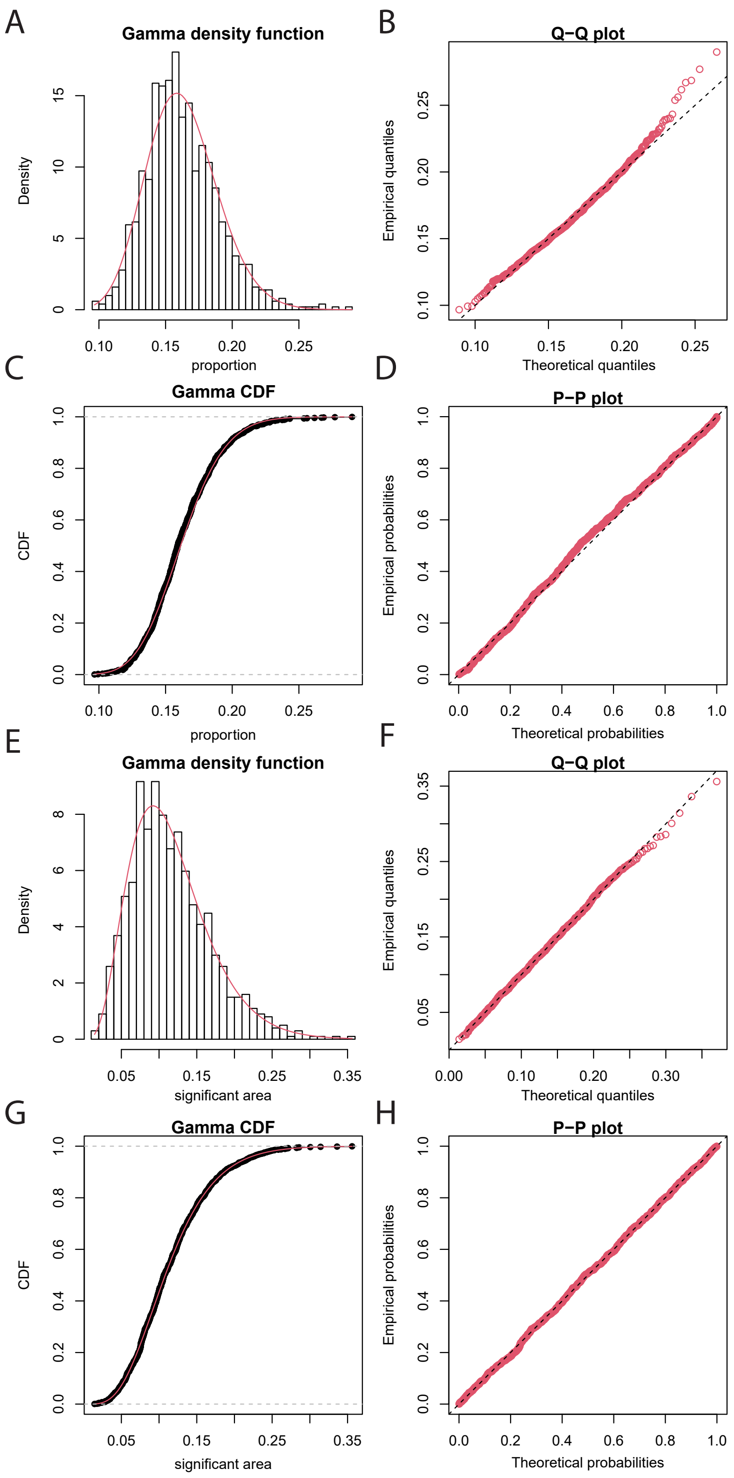

Fig. S3: Distribution characteristics of the significant area and proportion from 1000 sample permuted data fitted by a Gamma distribution. **A)** Histogram of the sample permutation distribution of the proportion with a Gamma density function overlaid. **B)** Q-Q plot showing the theoretical quantiles of the fitted Gamma distribution plotted against the empirical quantiles of the proportion from the sample permuted data. **C)** The fitted Gamma cumulative density function (CDF) plotted against the proportion. **D)** P-P plot of the theoretical probabilities of the fitted Gamma distribution against the Empirical probabilities of the proportion. **E)** Histogram of the sample permutation distribution of the significant area with a Gamma density function overlaid. **F)** Q-Q plot showing the theoretical quantiles of the fitted Gamma distribution plotted against the empirical quantiles of the significant area from the sample permuted data. **G)** The fitted Gamma cumulative density function (CDF) plotted against the significant area. **H)** P-P plot of the theoretical probabilities of the fitted Gamma distribution against the Empirical probabilities of the significant area. The distribution characteristics show that a Gamma distribution fits well with the CIBRA impact scores: significant area and proportion.

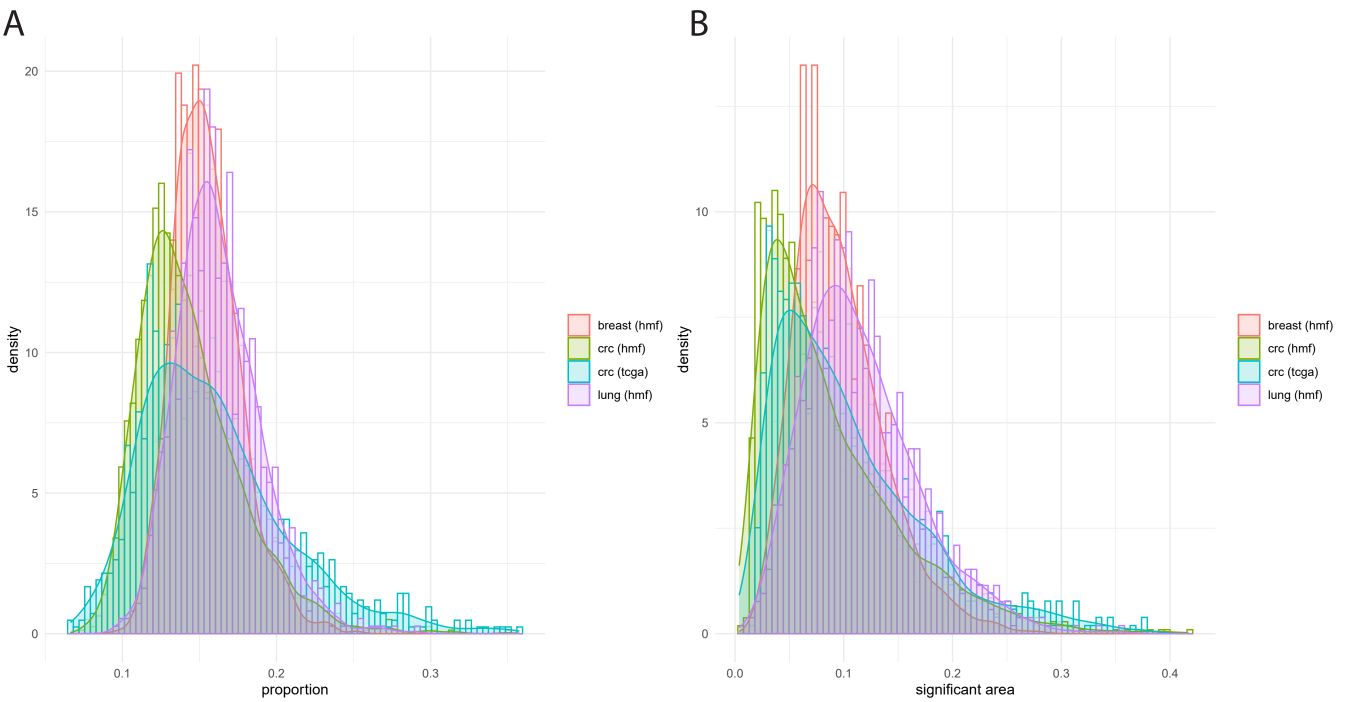

Fig. S4: Permutation distribution of the signal measures for different cancer types and datasets. **A)**Histogram overlaid with the density of the distribution for the proportion calculated for 1000 sample permutations for primary (crc_tcga) and metastatic colorectal (crc_hmf), breast (brca_hmf), and lung (luad_hmf) cancer. **B)** Histogram overlayed with the density of the distribution for the significant area calculated for 1000 sample permutations for primary (crc_tcga) and metastatic colorectal (crc_hmf), breast (brca_hmf), and lung (luad_hmf) cancer. Cancer type has an influence on the shape and position of the permutation distribution. The more heterogeneous the cohort, the wider the distribution. This is especially clear for the primary colorectal cancer dataset consisting of multiple stages of the disease. As such, generating the sample permutation distribution for each cancer type and dataset is advised.

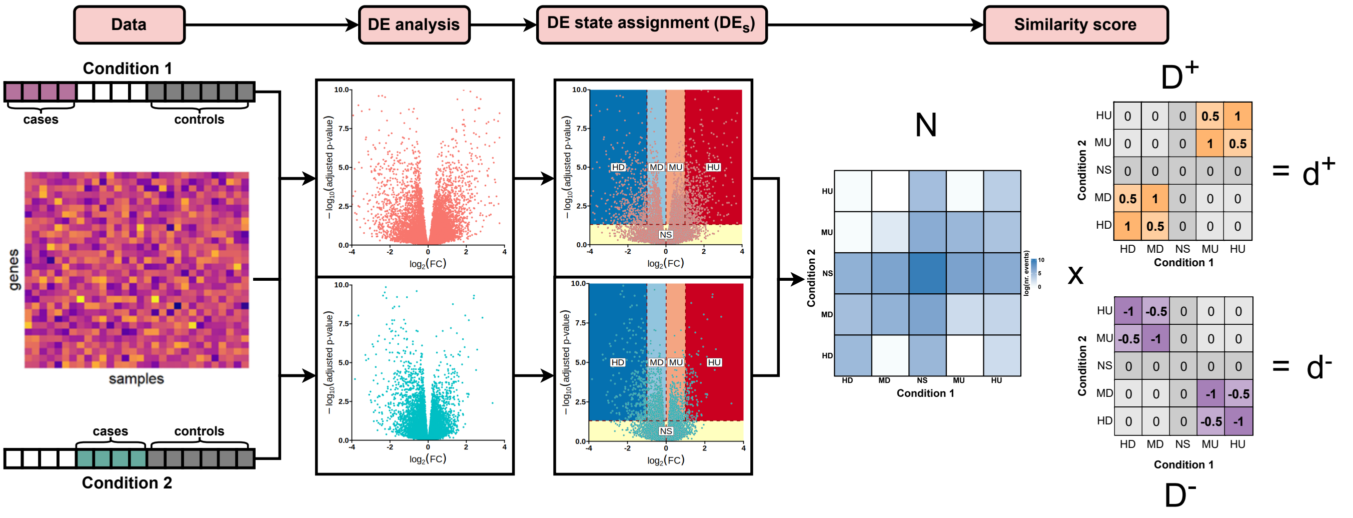

Fig. S5: Flowchart of the CIBRA similarity score calculation. Expression data and group definitions with shared controls are used to perform differential expression analysis (DE) similar to the impact score calculation. Using the generated log_2 fold changes (FC) and adjusted p-values, DE states (DE_s) are defined for each gene given the corresponding p-value and fold change for both conditions. To calculate the similarity between the two conditions, a contingency table (N) is generated between the DE_s of the two conditions. The frequency of the combination of DE_s is shown by a heatmap. A similarity (D^+) and anti-similarity (D^-) weight matrix is multiplied with the contingency table (N) to calculate the directional similarity scores d^+ and d^-. A permutation test is performed to estimate the significance of the d^+ and d^- scores.

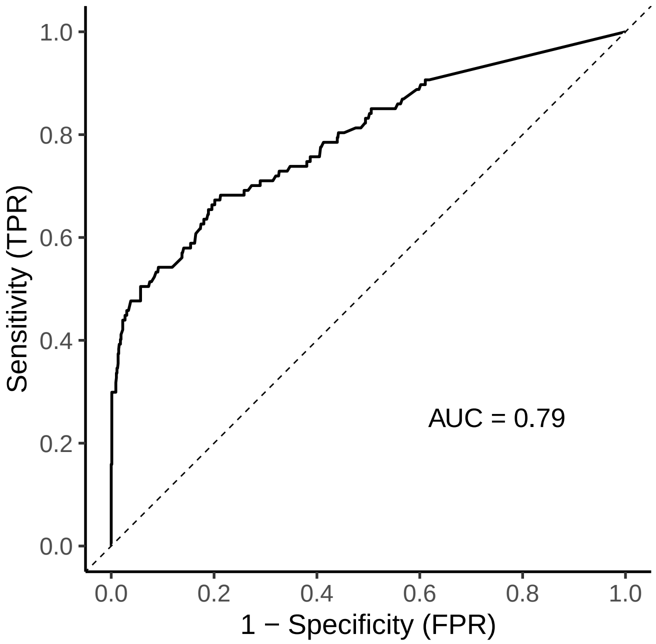

Fig. S6: Receiver operating characteristic (ROC) curve of the CIBRA impact score from the primary pan-cancer screen (TCGA) assessed against the list of testable genes from the CGC database. The area under the ROC curve (AUC) of the CIBRA impact score is 0.79.

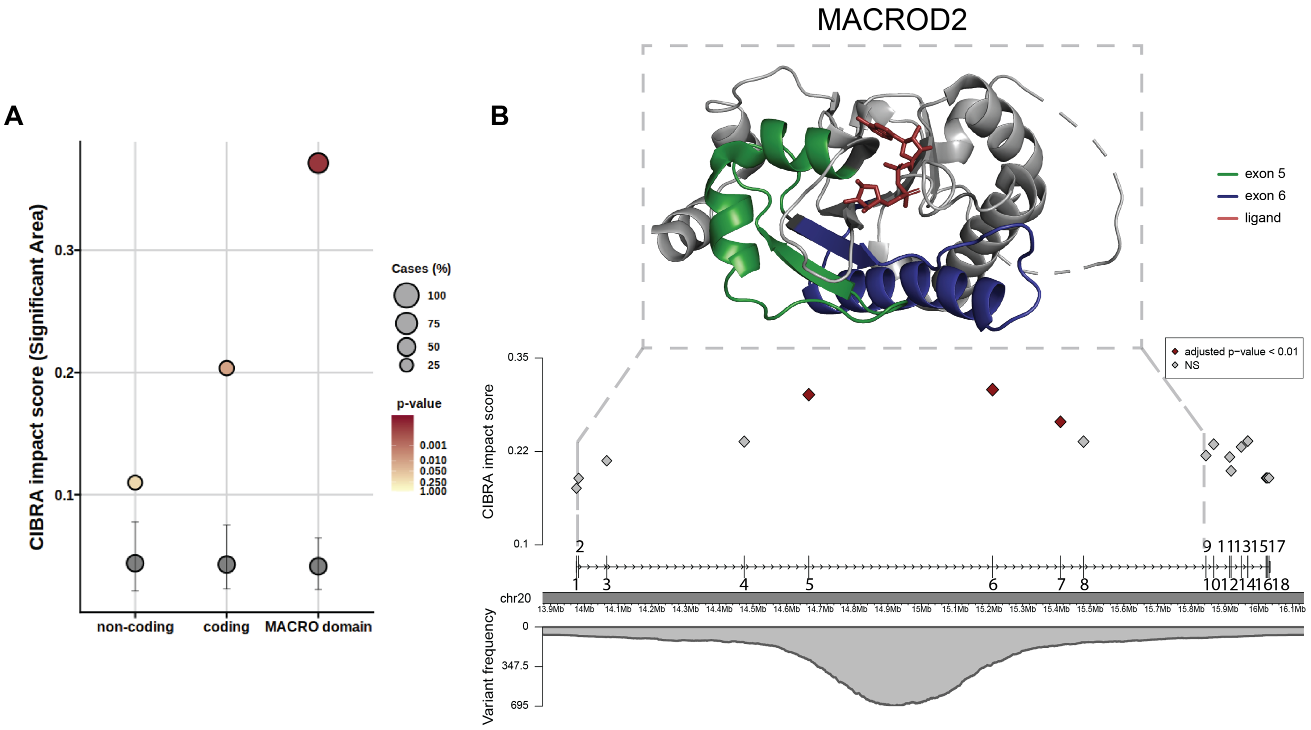

Fig. S7: Refining structural variants on genomic location in *MACROD2* for the identification of the most impactful subregion using CIBRA. **A)** CIBRA impact score visualization of metastatic microsatellite stable CRC samples with SVs affecting non-coding regions, the MACRO domain, or other coding regions within *MACROD2*. The gray dots represent the median permutation CIBRA score with the error bars representing the IQR. The color indicates the significance of the CIBRA score assessed by a permutation test. The dot size indicates the prevalence of the alteration (%). **B)** Overview of the CIBRA score per exon of coding SVs in *MACROD2* for microsatellite stable metastatic CRC. The top panel shows the crystallized protein structure (UniProt: 4iqy) colored by the exons with the highest CIBRA impact score, exon 5 (green) and exon 6 (blue). The ligand is colored in red. The middle panel shows the CIBRA impact score per assessed exon affected by SVs in *MACROD2* colored by significance. Red indicates significant (adjusted p-value < 0.01), and gray is not significant. A condensed transcript representation of *MACROD2* mapped to genome hg19 is shown, and the genome coordinates on chromosome 20 are represented with an ideogram. The lowest panel shows the SV frequency in 100-bp bins across 377 CRC samples.
