# Supplementary figures and images for: CIBRA identifies genomic alterations with a system-wide impact on tumor biology
Source: Bioinformatics. 2024 Sep 4;40(Suppl 2):ii37–44. doi: 10.1093/bioinformatics/btae384 (PMC11373315; doi:10.1093/bioinformatics/btae384)

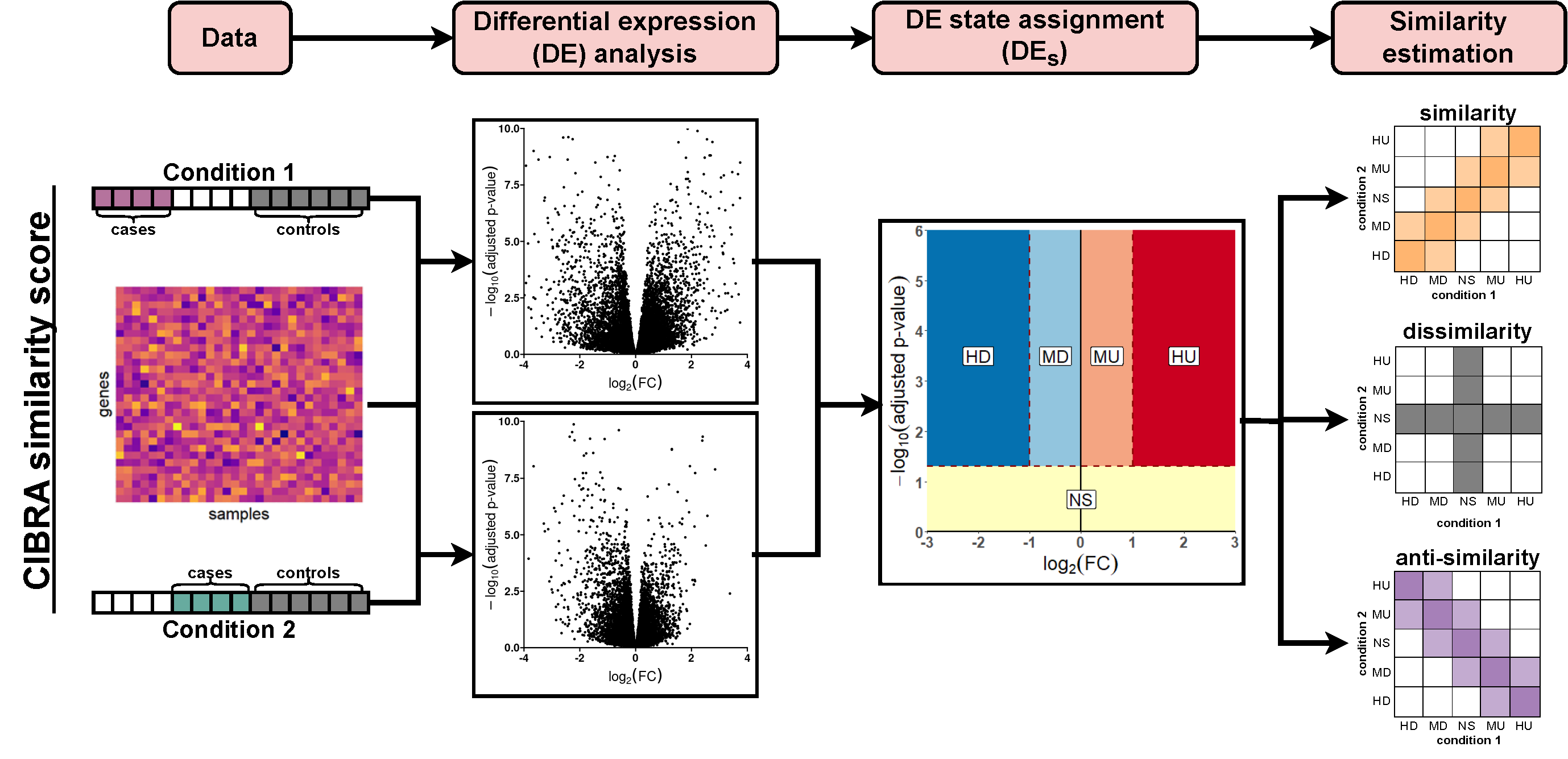

Supplement: btae384_Supplementary_Data [file btae384_supplementary_data.zip › CIBRA_flowchart_bottom (2).drawio.png]

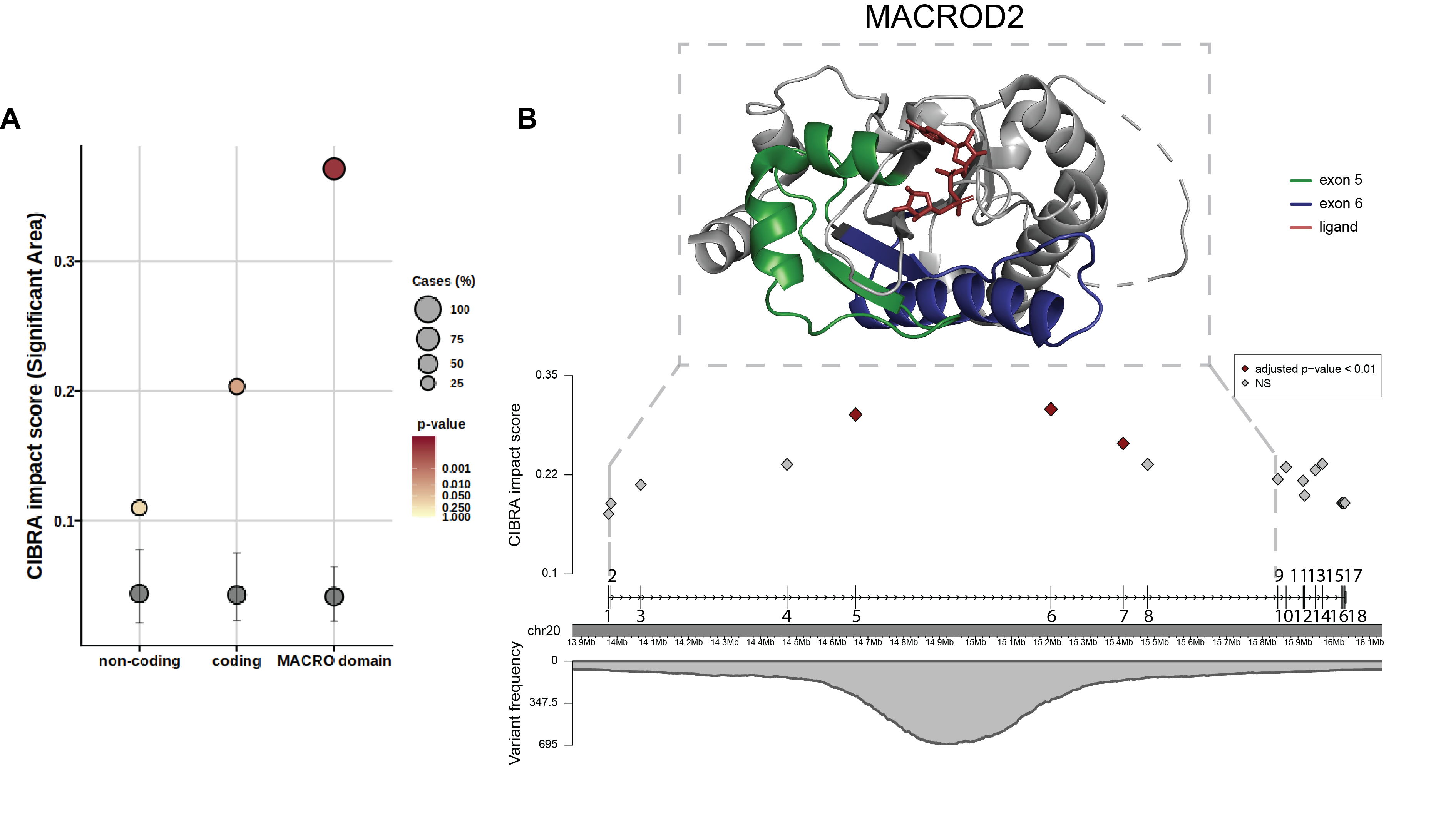

Supplement: btae384_Supplementary_Data [file btae384_supplementary_data.zip › MACROD2_MSS_HMF_location_screen.png]

invalid proportion case/control space

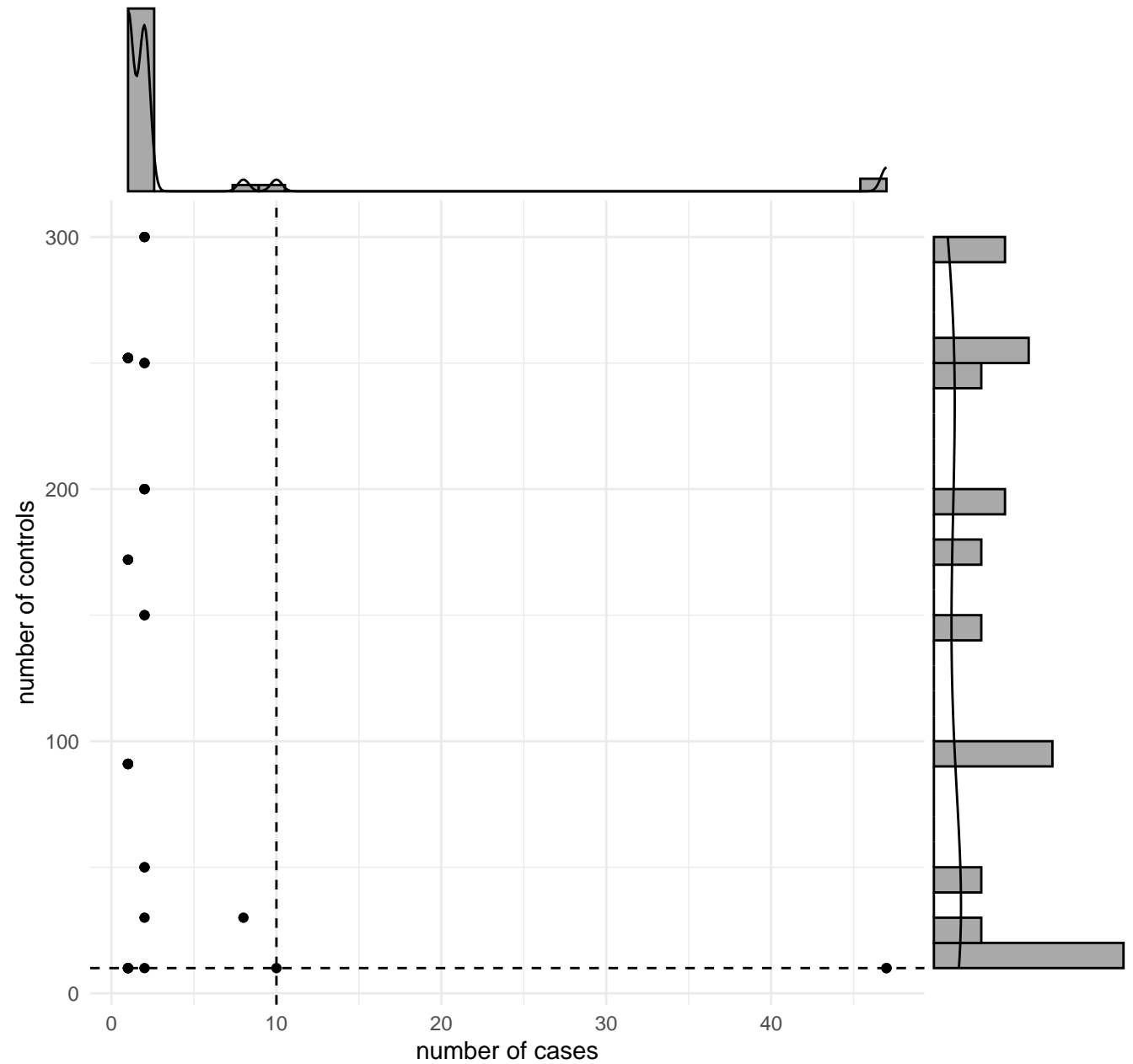

Supplement: btae384_Supplementary_Data [file btae384_supplementary_data.zip › supplemental_figure_1.pdf]

A

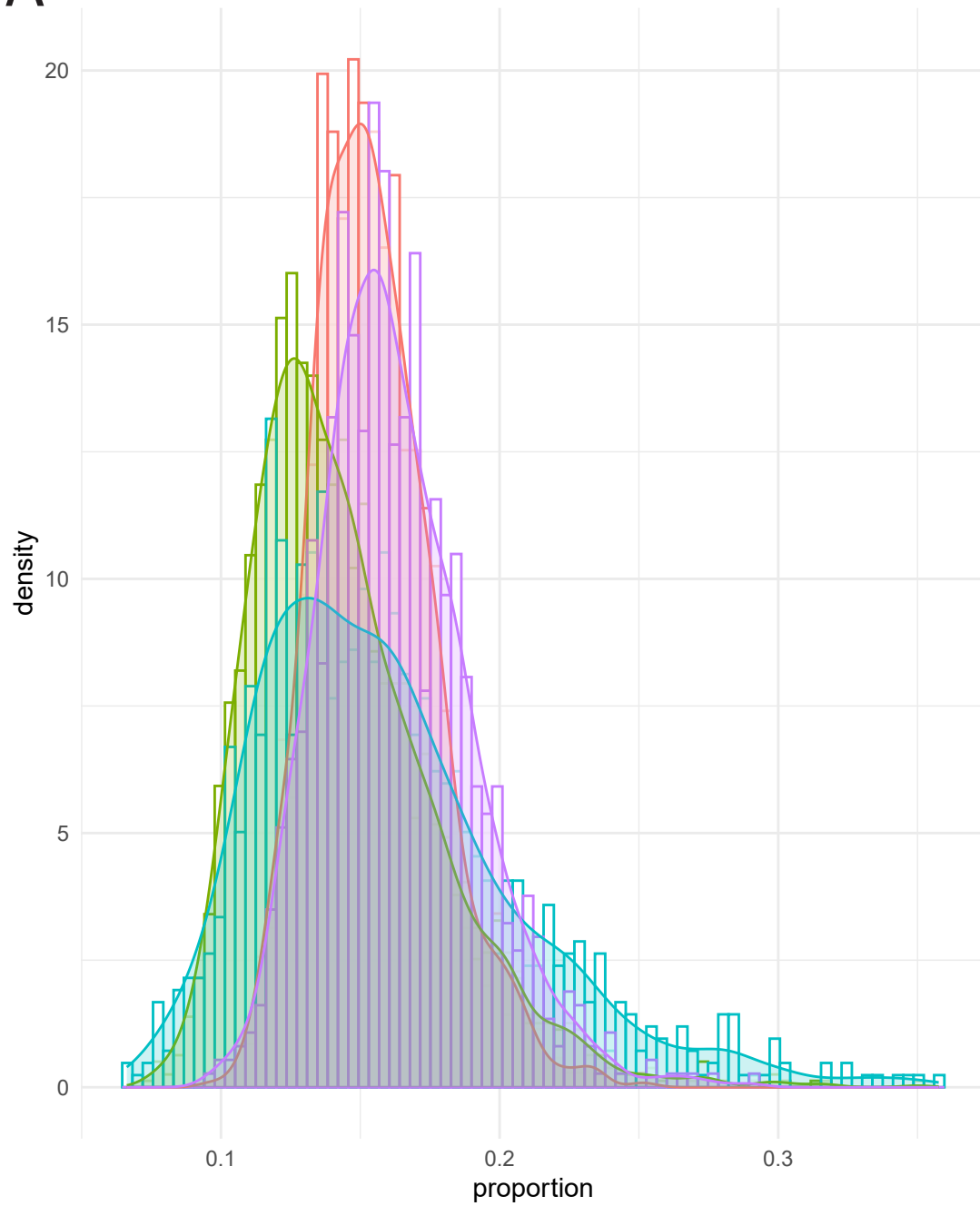

B

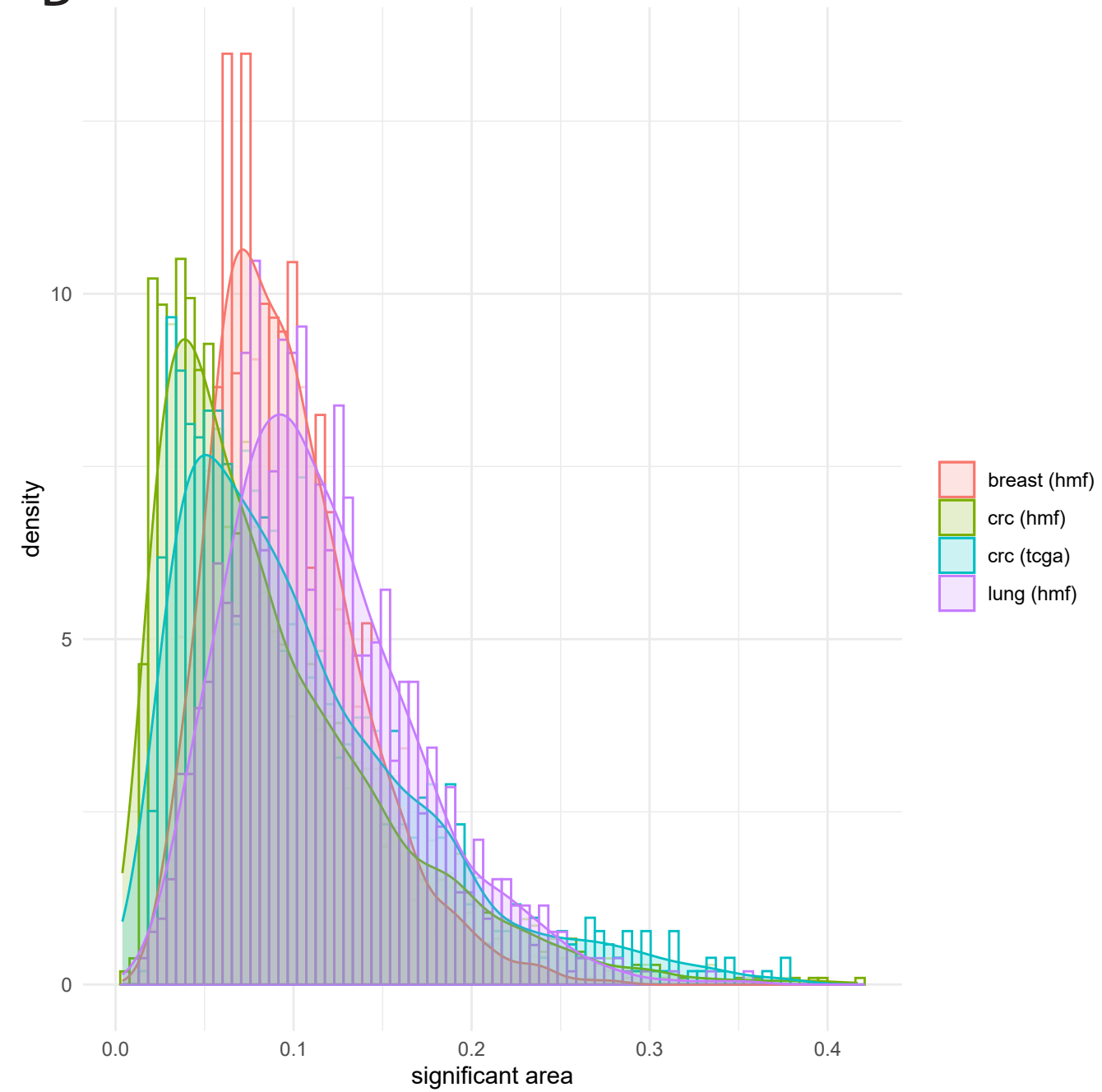

Supplement: btae384_Supplementary_Data [file btae384_supplementary_data.zip › supplemental_figure_3.pdf]

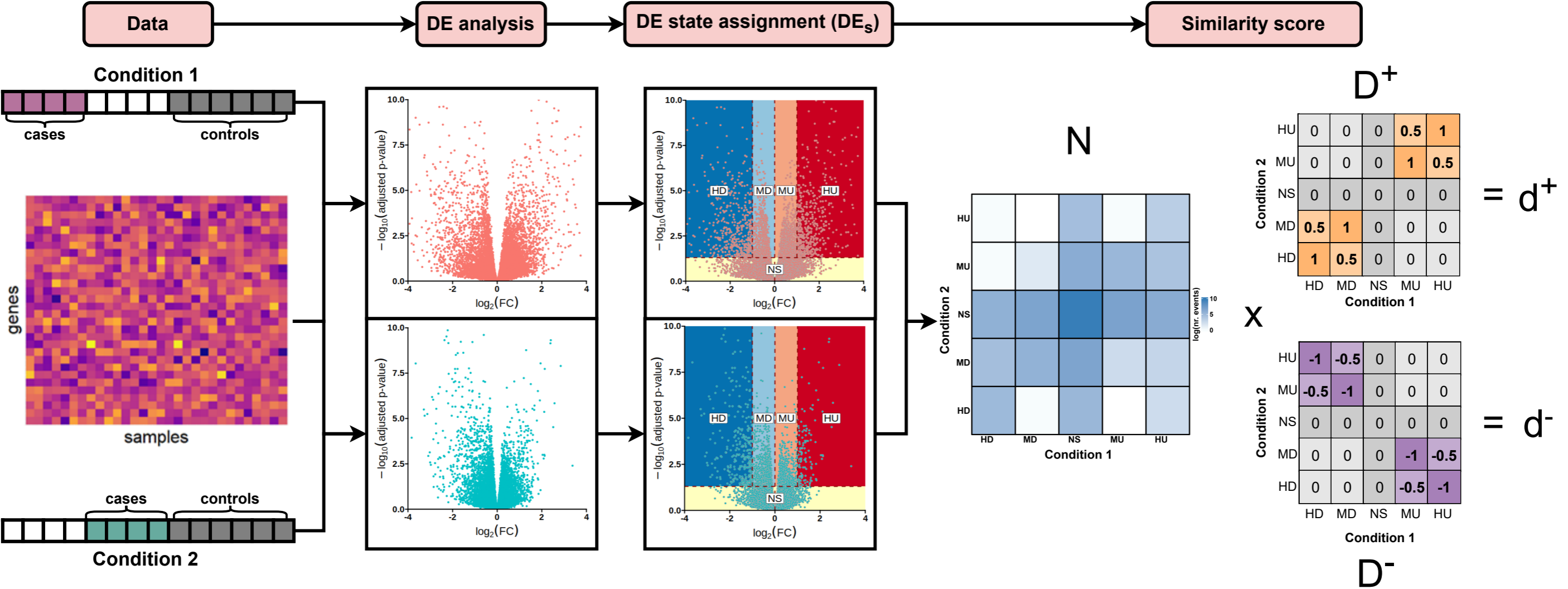

Supplement: btae384_Supplementary_Data [file btae384_supplementary_data.zip › supplemental_figure_4.pdf]

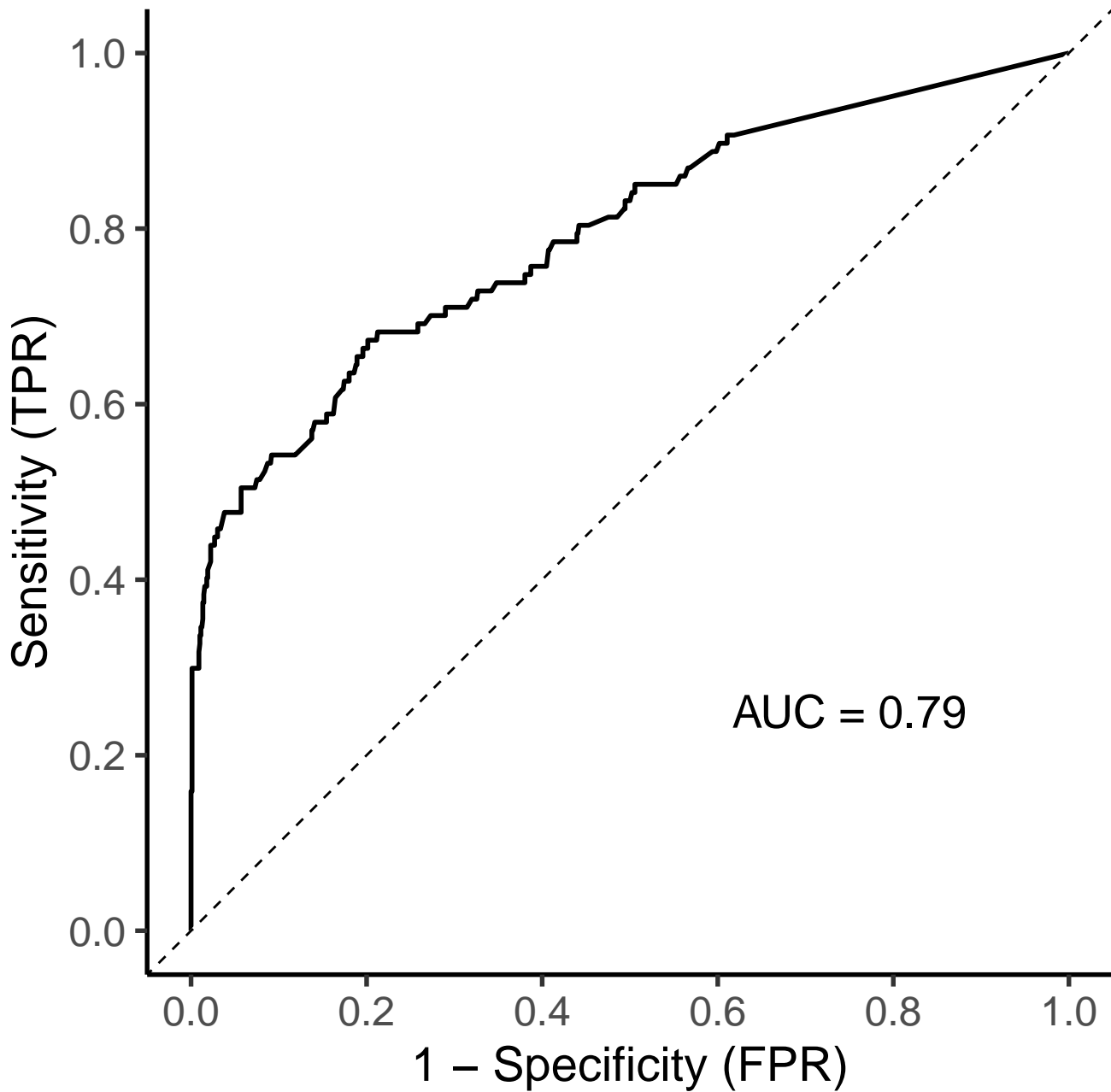

Supplement: btae384_Supplementary_Data [file btae384_supplementary_data.zip › supplemental_figure_5.pdf]
